# Rehmanniae Radix Praeparata in Blood Deficiency Syndrome: UPLC-Q-TOF-MS Profiling, Network Pharmacology, and PI3K-AKT Activation

**DOI:** 10.3390/ijms26083914

**Published:** 2025-04-21

**Authors:** Ruxi Gao, Fanyi Wang, Xiang Liu, Chu Yuan, Guoshun Shan

**Affiliations:** College of Pharmacy, Liaoning University of Traditional Chinese Medicine, Dalian 116600, China; gaoruxi1998@126.com (R.G.); 13233297672@163.com (F.W.); 15942632954@163.com (X.L.); y844833666@163.com (C.Y.)

**Keywords:** rehmanniae radix praeparata, blood deficiency syndrome, UPLC-Q-TOF-MS, network pharmacology, pharmacodynamics, PI3K-Akt signaling pathway

## Abstract

This study investigated the therapeutic mechanism of Rehmanniae Radix Praeparata (RRP) in treating blood deficiency syndrome (BDS) through integrated chemical analysis and pharmacological validation. UPLC-Q-TOF-MS identified chemical components of Rehmanniae Radix (RR) and RRP, with network pharmacology analysis suggesting AKT1 and NOS3 in the PI3K-AKT pathway as potential therapeutic targets. Pharmacodynamic evaluations using ELISA, hematological analysis, histopathology, and immunohistochemistry demonstrated RRP’s efficacy in improving hematological parameters, energy metabolism, and organ pathology in BDS mice. Experimental validation via RT-qPCR and Western blot confirmed significant upregulation of AKT1 and NOS3 mRNA and protein expression following RRP treatment. The findings indicate that RRP alleviates BDS by activating the PI3K-Akt signaling pathway to modulate AKT1 and NOS3 expression, providing mechanistic insights into its therapeutic actions.

## 1. Introduction

Blood deficiency syndrome (BDS), previously referred to as hematopoietic–metabolic dysregulation syndrome (HMDS) in clinical diseases, is a pathological state characterized by impaired hematopoietic function coupled with systemic metabolic imbalance. Unlike the classical TCM concept of blood deficiency, which focuses on insufficient blood nourishment, HMDS encompasses dual dysregulation of hematopoiesis (e.g., erythroid suppression) and metabolic homeostasis (e.g., energy metabolism disorders), typically manifested as abnormal nutrient utilization, and multi-organ dysfunction. This syndrome shows clinical parallels with modern disease entities, including chemotherapy-induced myelosuppression, chronic inflammatory anemia, and metabolic anemia associated with chronic kidney disease [1,2]. The diagnostic hallmark extends beyond simple hemoglobin reduction to include dysregulated hematopoietic markers (e.g., abnormal erythropoietin levels) and metabolic indicators (e.g., altered cAMP/cGMP ratios) [3]. Unlike anemia, which is defined by hemoglobin concentration reduction, HMDS involves comorbid hematopoietic–metabolic dysregulation—while causing splenomegaly and circulatory impairment like traditional BDS, it additionally disrupts mitochondrial energy metabolism and nutrient sensing pathways [4], leading to multi-system complications.

A search of the MPNS website [mpns.science.kew.org (URL: 16 March 2023)] revealed that Rehmanniae Radix is the root tuber of the plant *Rehmannia glutinosa* Libosch, primarily produced in Henan, Shandong, and other locations. Rehmanniae Radix (RR) is a commonly utilized Chinese medicine in clinical practice, known for its properties of reducing inflammation, lowering body temperature, and promoting fluid replenishment; it is primarily employed for conditions characterized by fever, skin eruptions, hematemesis, and similar symptoms. Rehmannia Radix Praeparata (RRP) is a processed dried root tuber of *Rehmannia glutinosa* Libosch. It functions to promote fluid replenishment, enhance blood production, support bone marrow health, and strengthen the lower back and knees, and it is primarily utilized for conditions such as fluid deficiency, anemia, lumbar weakness, and fatigue-related bone disorders. The suboptimal living habits of modern populations have led to the emergence of the BDS population. This has resulted in increasing clinical demand for RRP. However, current research on RRP has predominantly focused on its clinical applications, while the material basis and mechanism of its effects have not been thoroughly investigated.

Network pharmacology and pharmacodynamics are commonly employed to elucidate the mechanisms underlying TCM. Consequently, RRP should be utilized to treat BDS. Network pharmacology and pharmacodynamics were employed to study the mechanisms by which RRP treats BDS. Initially, UPLC-Q-TOF-MS was utilized to analyze the differential components between RR and RRP. Subsequently, based on the differential components obtained from the analysis, a network pharmacology method was established to explore the mechanism of action of RRP in the treatment of BDS from the perspective of targets and pathways. The pharmacodynamics of RRP were evaluated using ELISA, routine blood tests, pathological sectioning, and immunohistochemistry. RT-qPCR and Western blotting were performed to verify the pathways and targets involved. In this study, a combination of network pharmacology and pharmacodynamics was employed to elucidate the mechanism of RRP in the treatment of BDS. These findings are expected to provide a theoretical foundation for elucidating the mechanism of action of RRP in the treatment of BDS.

## 2. Results

### 2.1. Analysis of Chemical Constituents

#### 2.1.1. Identification of Chemical Constituents of RR and RRP

**Figure 1:**
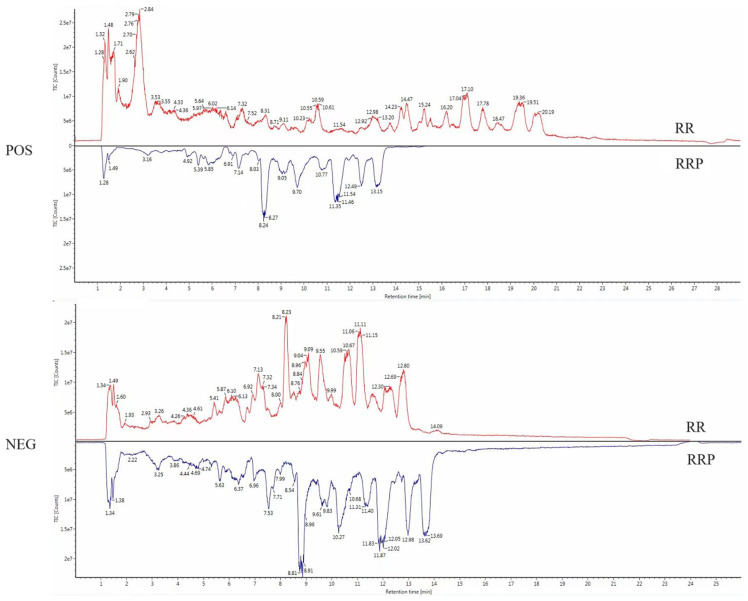
UPLC-Q-TOF-MS total ion chromatograms of RR and RRP (POS is a positive ion particle flow graph, NEG is a negative ion particle flow graph).

UPLC-Q-TOF MS technology was employed to analyze the chemical constituents of RR and RRP in the positive and negative ion collection modes. The binary-contrast TIC diagrams in the positive and negative ion modes are presented in Figure 1.

The chemical constituents of *Rehmannia glutinosa* and its plants of the same family and genus were identified, and a database of chemical constituents of *Rehmannia glutinosa* (containing 218 components) was established after consulting the literature. The methanol extracts of the RR and RRP extracts were analyzed using the UNIFI platform, and the compounds were identified by combining the retention times and accurate relative molecular masses of the compounds.

#### 2.1.2. Identification of the Chemical Constituents of *Rehmannia glutinosa*

**Figure 2:**
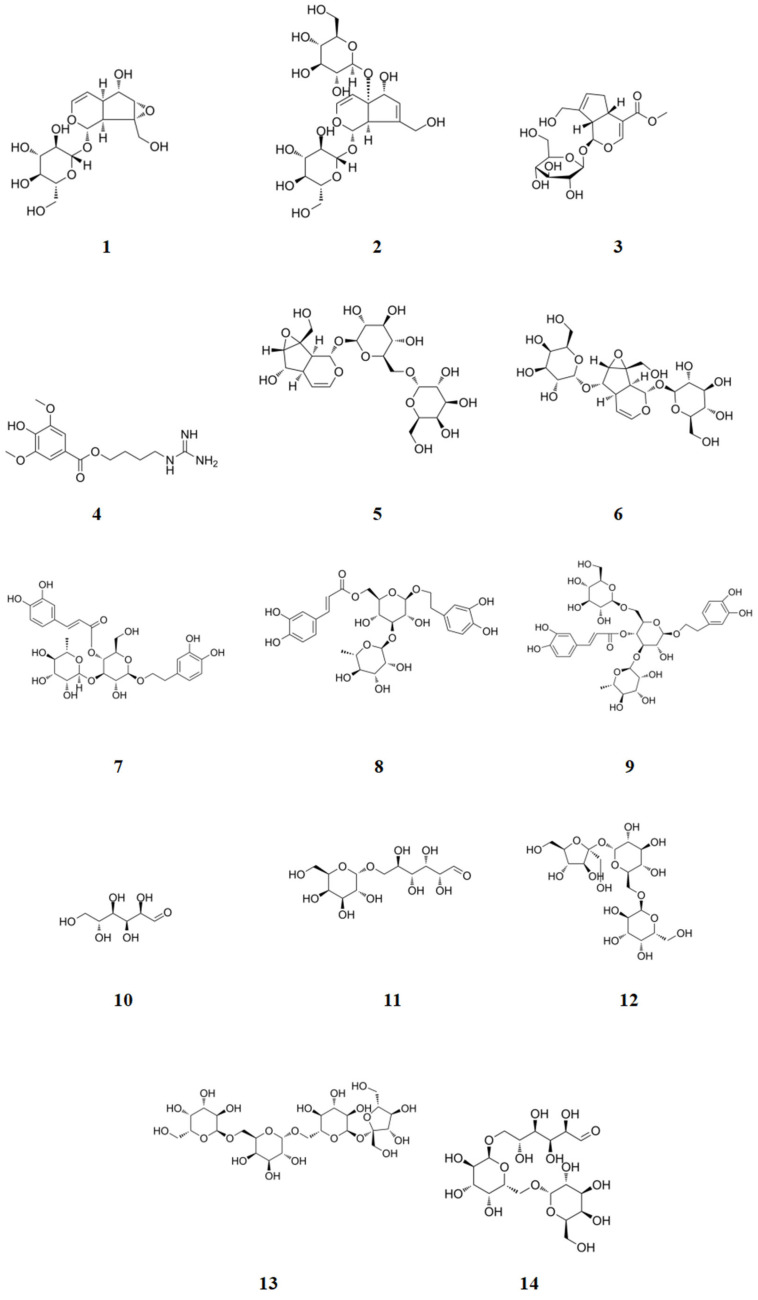
The main chemical components of *Rehmannia glutinosa*.

**Figure 3:**
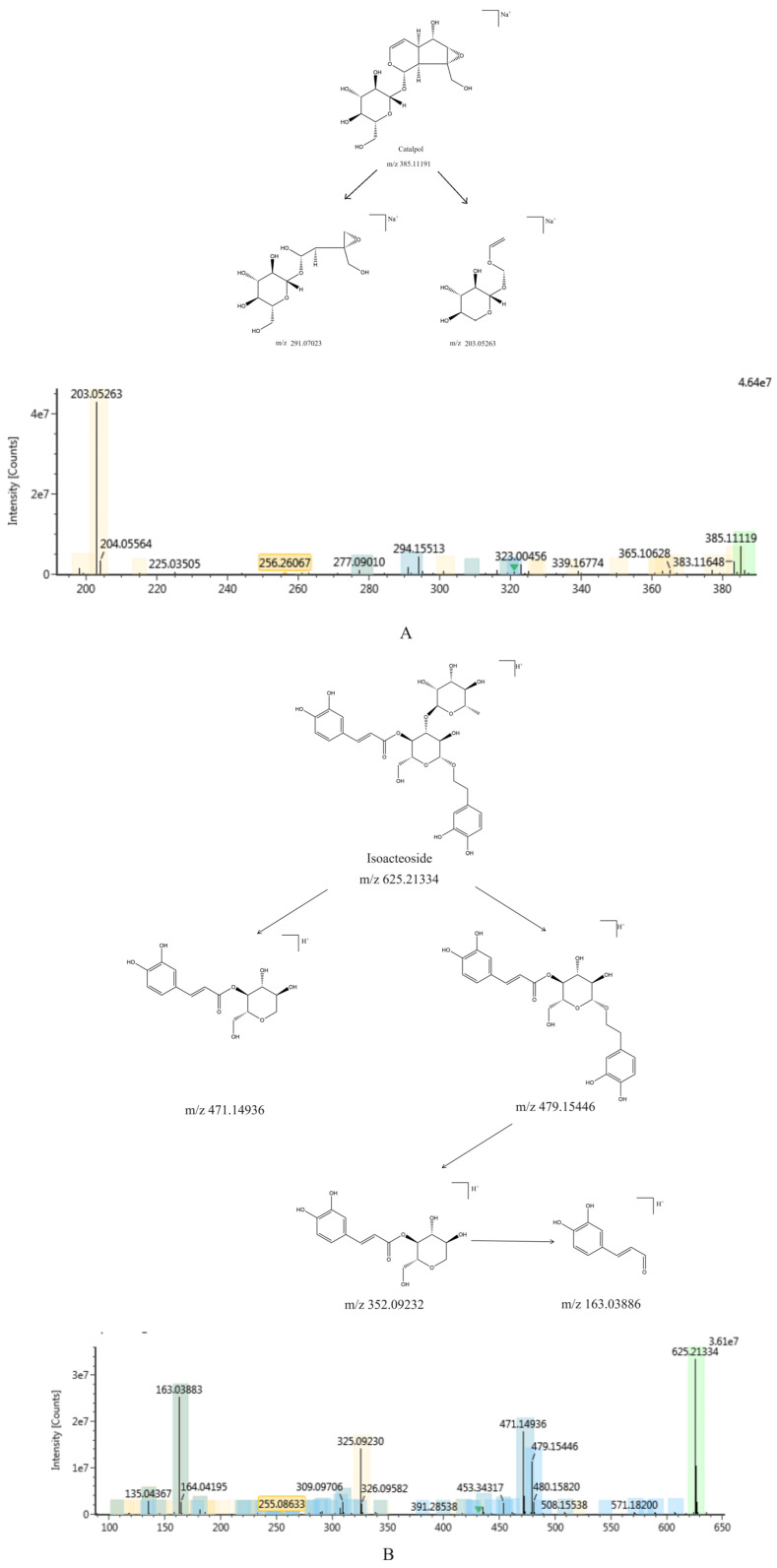

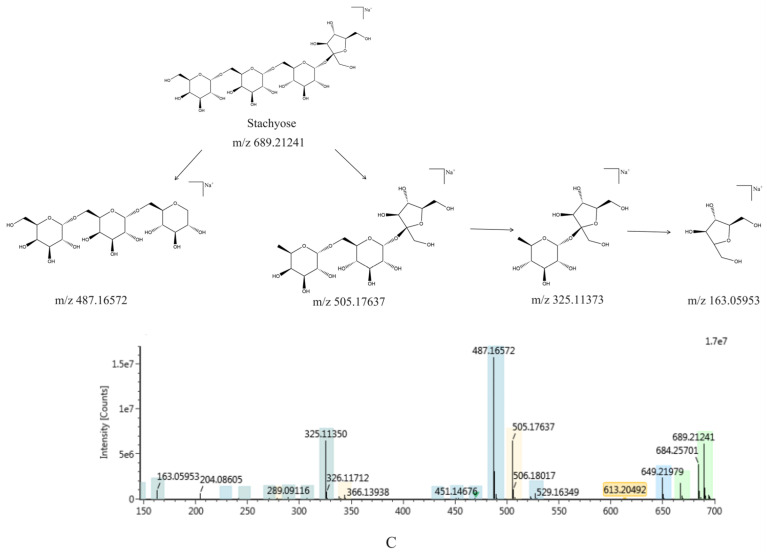
Cracking law of chemical constituents of *Rehmannia glutinosa* ((**A**) is catalpol, (**B**) is isoacteoside, and (**C**) is stachyose). The bar graph is the corresponding mass spectrum of the fragmentation rule, the abscissa is *m*/*z*, and the ordinate is the response value. (*m*/*z* is mass-to-nucleus ratio, and the upper right corner of the molecular formula is the additive ion).

The iridoid compounds identified in *Rehmannia glutinosa* were primarily catalpol, milletoside, geniposide, leonuride, rehmannioside A, and rehmannioside B. The compound information is presented in Table 1 (**1**–**6**) and the corresponding chemical structures are illustrated in Figure 2 (**1**–**6**). This class of components exhibits a characteristic dihydropyran ring cis-connected unit structure of a five-membered ring, and there are two main types of cyclopentane and secoiridoid glycosides [5]. This type of component readily loses sugar (-C_6_H_10_O_5_) in secondary mass spectrometry to produce aglycone, which continues to fragment. Due to the hemiacetal structure and active chemical properties of secoiridoid glycosides, reverse Diels-Alder (RDA) cleavage occurs after glycosidic bond cleavage, resulting in the loss of fragment ions such as H_2_O, CO_2_, and CH_3_ [6]. The cleavage pathway, using catalpol as an example, is illustrated in Figure 3A. In the positive ion mode, a quasi-ion peak [M + Na]^+^ was detected with an *m*/*z* of 385.1119. At high collision energies, two fragmentation pathways may occur: loss of -H_2_O and -CH_2_-C=C fragments to obtain fragment ions with *m*/*z* 291.07023, and after glycosidic bond cleavage, RDA cleavage occurs to obtain fragment ions with *m*/*z* 203.05263.

The phenylethanoid glycosides in *Rehmannia glutinosa* primarily consist of verbascoside, isoacteoside, and echinacoside. The compound information is presented in Table 1 (**7**–**9**), and the corresponding chemical structures are illustrated in Figure 2 (**7**–**9**). The molecular structure of phenylethanoid glycosides typically comprises four components: phenylethanone, glucose, rhamnose, and phenylpropenylphthalide. Its structural characteristic is that β-glucopyranose serves as the parent nucleus, and substituted phenylethyl and cinnamoyl groups are connected via ester bonds and oxygen glycoside bonds [7]. Utilizing isoacteoside as an exemplar, the cleavage pathway is depicted in Figure 3B. A quasi-ion peak [M + H]^+^, *m*/*z* 625.21334, was detected. There may be two pathways for high collision energy: the *m*/*z* of the fragment ion obtained by removing the phenylpropanoid group was 471.14936, and the glycosyl group was removed twice consecutively to obtain fragment ions with *m*/*z* values of 352.09232 and 163.03886. After removing the glycosyl group, the fragment ion *m*/*z* was 479.15446, the phenylpropanoid group was removed to obtain a fragment ion *m*/*z* of 352.09232, and the glycosyl group was removed to obtain a fragment ion *m*/*z* of 163.03886.

The sugars in *Rehmannia glutinosa* predominantly consist of glucose, fructose, galactose, sucrose, melibiose, raffinose, stachyose, and mannose. The compound information is presented in Table 1 (**10**–**14**), and the corresponding chemical structures are illustrated in Figure 2 (**10**–**14**). The oligosaccharides, trisaccharides, and disaccharides in *Rehmannia glutinosa* undergo continuous hydrolysis into monosaccharides during processing. The cracking pathway of stachyose is depicted in Figure 3C. A quasi-ion peak [M + H]^+^ (*m*/*z* 689.21241) was detected. At high collision energy, fructose may be removed, resulting in a fragment ion with an *m*/*z* of 487.16572; a fragment ion with an *m*/*z* of 505.17637 was obtained by losing a 1,6-glycosidic bond, a fragment ion with an *m*/*z* of 325.11373 was obtained by losing a-galactose, and a fragment ion with an *m*/*z* of 163.05953 was obtained by losing a-galactose.

#### 2.1.3. Comparative Analysis of the Chemical Constituents of *Rehmannia glutinosa* Before and After Processing

**Figure 4:**
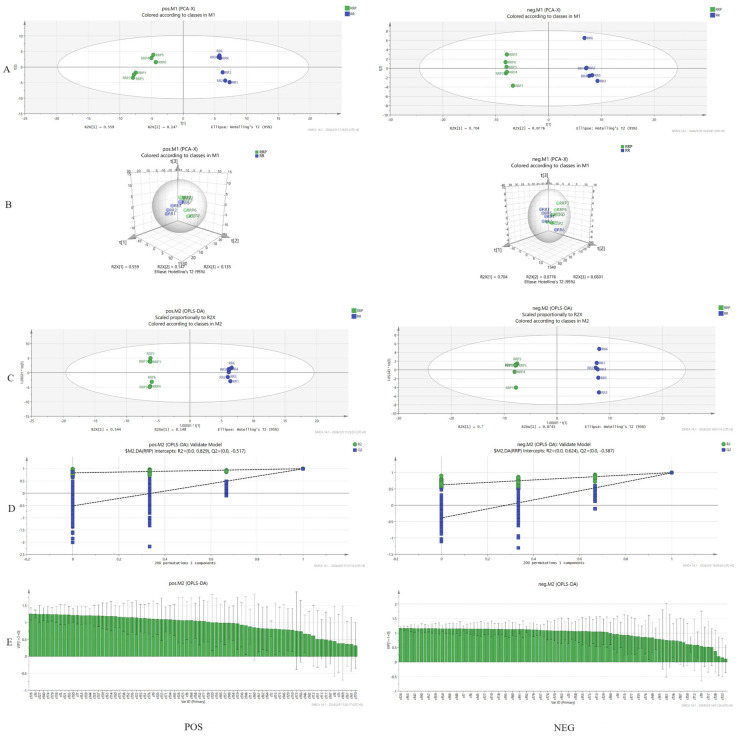
Multivariate statistical analysis ((**A**) PCA, (**B**) PCA 3D score, (**C**) OPLS-DA, (**D**) fiducial distribution, and (**E**) vip value) (POS is positive ion analysis and NEG is negative ion analysis).

The LOG pretreatment method was employed to analyze the raw and prepared rehmannia. According to the results of PCA [8], in the positive ion mode, the model test R_2Xcum_ = 0.887, Q_2cum_ = 0.613; in the negative ion mode, the model test R_2Xcum_ = 0.841, Q_2cum_ = 0.615, indicating that the PCA model exhibits good stability and predictive reliability. These scores are presented in Figure 4A. A three-dimensional (3D) score plot is illustrated in Figure 4B. Each sample was concentrated in the corresponding area and could be clearly differentiated, suggesting that there were discernible differences in the chemical compositions of the raw and prepared rehmannia.

Based on principal component analysis (PCA), orthogonal partial least squares discriminant analysis (OPLS-DA) was performed on the RR and RRP, and the model was identified and verified through internal and external validation. In the positive ion mode, the internal validation result was RR-RRP (R_2_Y = 0.999, Q_2_ = 0.994), indicating that the model exhibited strong predictive capability. The external validation results were RR-RP (R_2_ = 0.829, Q_2_ = −0.517) (Figure 4C). In Figure 4D, Q_2_ < 0, and all Q_2_ points consistently remained below the original blue Q_2_ points from left to right, suggesting that the evaluation model was reliable and effective, with no evidence of overfitting [9]. In the negative ion mode, the internal validation result was RR-RRP (R_2_Y = 0.999, Q_2_ = 0.992), demonstrating the model’s robust predictive ability. The external validation results were RR-RR (R_2_ = 0.624, Q_2_ = −0.387). In the figure, Q_2_ < 0, and all Q_2_ points consistently remained below the original blue Q_2_ points from left to right, indicating that the evaluation model was reliable and effective, with no evidence of overfitting. The score plot and permutation test results are presented in Figure 4E. The score plot reveals that within the 95% confidence interval, each group can be clearly differentiated into two categories, suggesting that processing has a significant effect on the chemical composition between the groups.

With respect to positive ions, we detected 95 active ingredients and screened 59 components whose expression changed after processing. The main compounds were iridoids, phenylethanoid glycosides, carbohydrates, benzaldehydes, phenylpropanoid glycosides, organic acids, dihydroflavonoids, 5-hydroxymethylfurfural, and their derivatives. There were 32 types of components with greater content in RR and 27 types of components with greater content in RRP.

In the negative ion mode, we detected 79 components and screened 39 components that changed after processing. These compounds include iridoids, phenylethanoid glycosides, 5-hydroxymethylfurfural, and its derivatives. There were 19 types of components with greater contents in RR and 20 types of components with greater contents in RRP. Components with a VIP value > 1 were screened as differential components. The related information for the compounds identified in the positive and negative ion modes is shown in Table 2 and Table 3.

### 2.2. Results of Network Pharmacology Analysis

#### 2.2.1. Target Analysis of Rehmanniae Radix Praeparata and Blood Deficiency Syndrome

**Figure 5:**
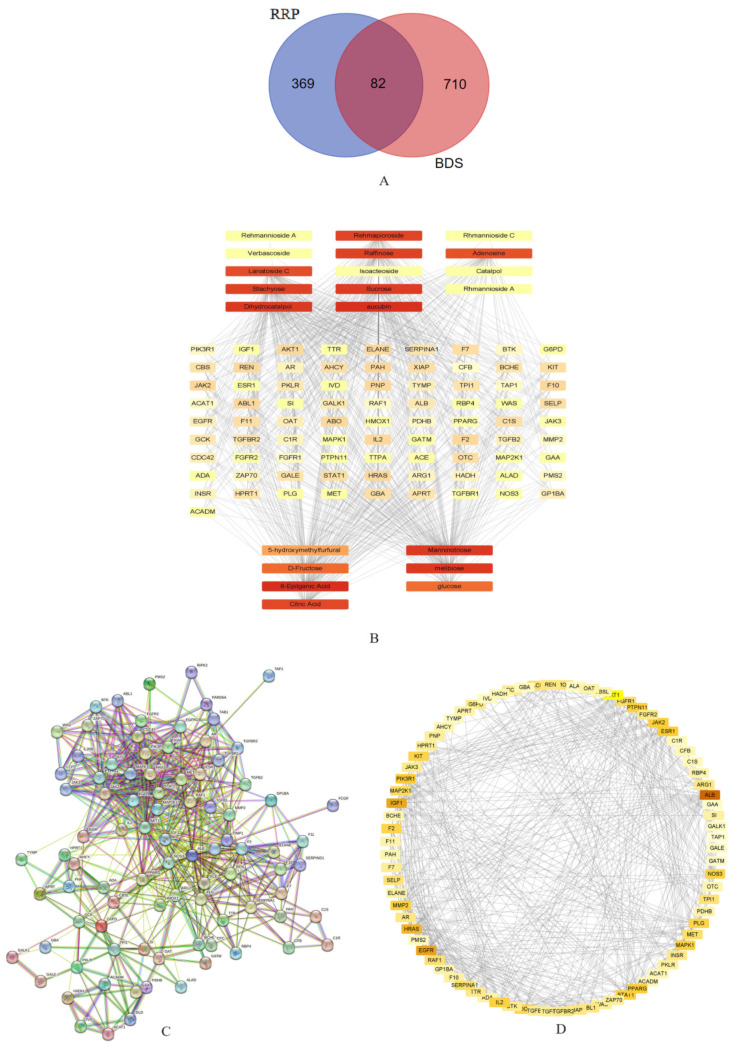
Screening of core targets ((**A**) Venn diagram of RRP and BDS intersection targets. (**B**) “Component-target” network diagram, (**C**) PPI network diagram of intersection targets, and (**D**) Intersection target network diagram).

Network pharmacological analysis was performed on the screened differential components. A total of 369 potential protein targets were screened using differential components on the four websites. In total, 710 potential protein targets were screened for BDS. Among these, 82 common targets were identified in the component and disease categories (Figure 5A).

#### 2.2.2. Construction of a “Component-Target” Network

As in previous research, the components that decreased in content after processing included raffinose, sucrose, dihydrocatalpol, aucubin, stachyose, adenosine, lanatoside C, isoacteoside, verbascoside, rehmapicroside, catalpol, romannioside C, rehmannioside A, and romannioside A; the group whose content increased after processing included D-fructose, glucose, 5-hydroxymethylfurfural, melibiose, citric acid, 8-epiloganic acid, and mannotriose. Through Cytoscape 3.8.0, a “component-target” network for RRP treatment of BDS was constructed. The Network Analyzer tool of the software is used to calculate the network topology parameters. The results are shown in Figure 5B.

#### 2.2.3. Construction of Protein–Protein Interaction (PPI) Networks

The STRING database and Cytoscape 3.8.0 were used to screen the key targets. Fourteen core targets were identified: EGFR, STAT1, HRAS, PPARG, HMOX1, IGF1, AKT1, F2, PLG, NOS3, ALB, IL2, TPI1, and SERPINA1 (Figure 5C,D).

#### 2.2.4. Kyoto Encyclopedia of Genes and Genomes and Gene Ontology Enrichment Analysis

**Figure 6:**
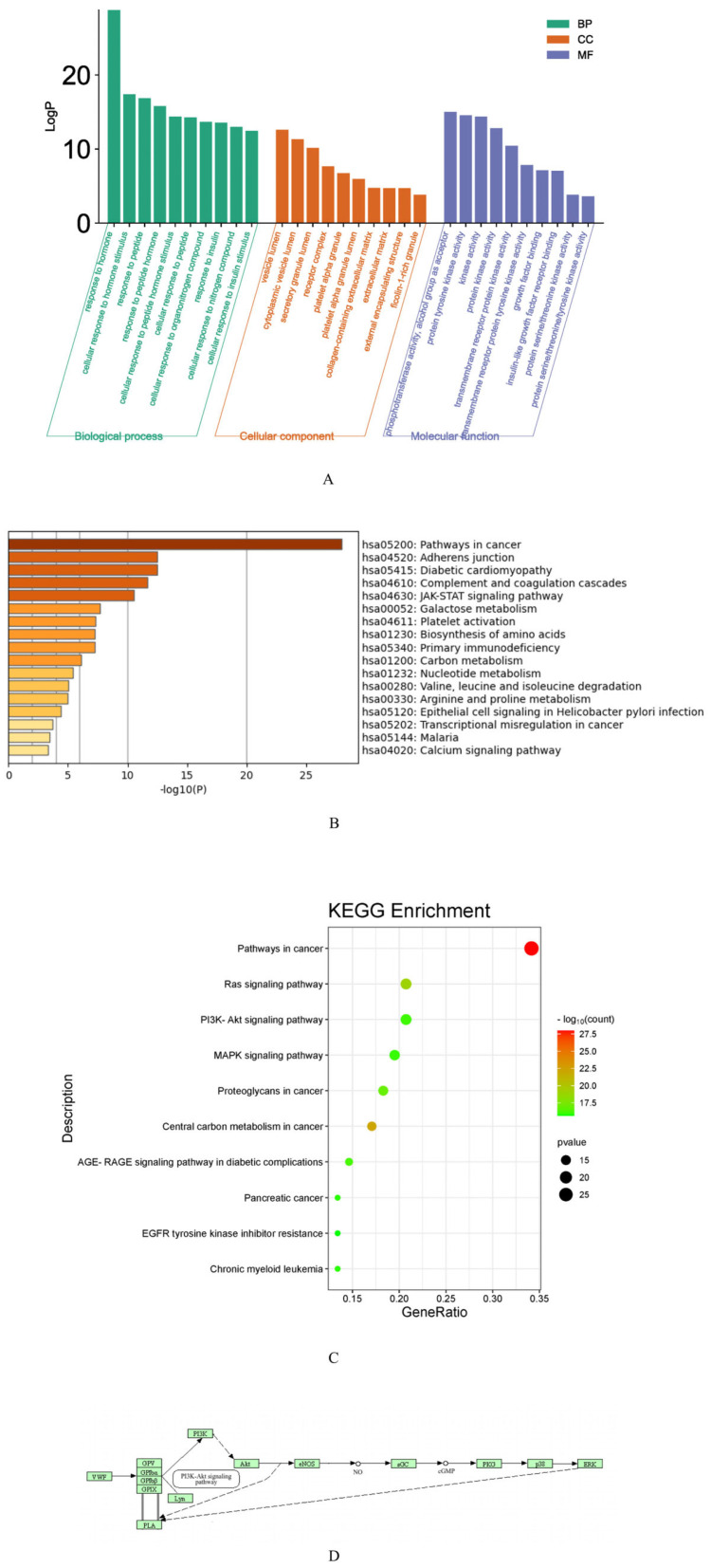
Network pharmacology pathway screening ((**A**) GO function enrichment analysis histogram, (**B**) KEGG enrichment pathway results, (**C**) KEGG enrichment pathway bubble chart, and (**D**) hsa04611 pathway).

The potential targets of RRP for BDS were analyzed using the Metascape database. A filter criterion of *p* < 0.05 was established. Subsequently, for the GO enrichment analysis, the top 10 biological process (BP), cell composition (CC), and molecular function (MF) pathways were selected (Figure 6A). The analysis results indicated that the potential targets were primarily associated with hormone response, gland development, cell population proliferation, nutrient level response, wound response, small molecule catabolic processes, hematopoiesis, and other pathways. KEGG pathway enrichment analysis was conducted on the pathways with the top 17 LogP values. The top 10 pathways were selected to generate an enriched bubble chart (Figure 6B,C). The results demonstrated that the potential targets were predominantly related to adhesion junctions, diabetic heart disease, complement and coagulation cascades, the JAK-STAT signaling pathway, galactose metabolism, platelet activation, amino acid biosynthesis, and other pathways.

Combined with the pathogenesis of blood deficiency syndrome, the possible pathway involved was identified as hsa04611 (platelet activation) (Figure 6D). This pathway can directly regulate the synthesis and release of cGMP. It was speculated that the targets acting on the PI3K-Akt signaling pathway are AKT1 and NOS3, which were synthesized as the key targets obtained in the “component-target” analysis.

#### 2.2.5. Molecular Docking

**Figure 7:**
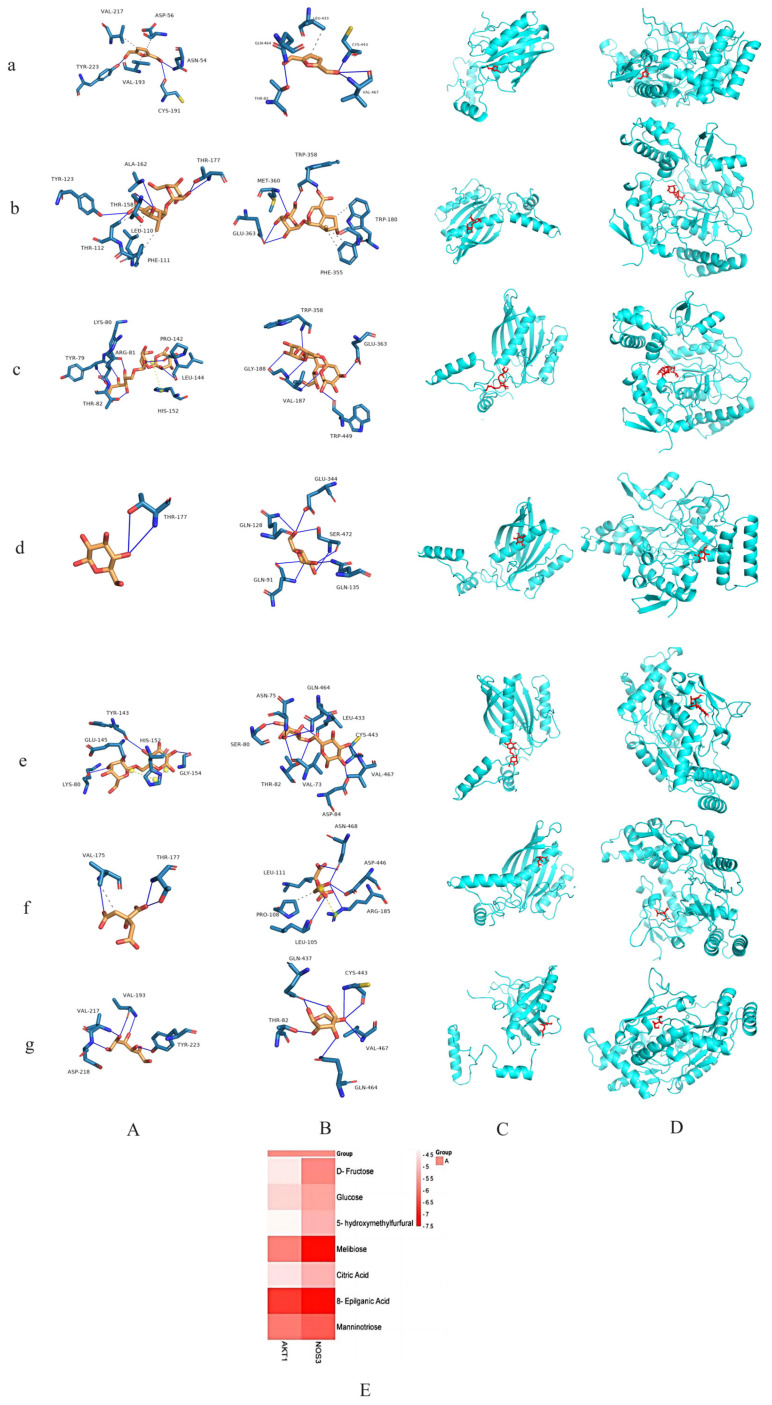
Molecular docking. (**A**) Docking of AKT1 to its active ingredients. (**B**) Docking of NOS3 to its active ingredients. (**C**) The optimal conformation of AKT1 with the active ingredient. (**D**) The optimal conformation of NOS3 with the active ingredient. (**E**) Combined energy heat maps where a is 5-hydroxymethylfurfural, b is 8-epiloganic acid, c is manninotriose, d is glucose, e is melibiose, f is citric acid, and g is D-fructose. Residue labels (e.g., ALAH, VAL-13) indicate amino acid positions in the protein binding pocket. Numbers (e.g., TIM: 177, FAP:160) represent bond angles or distances (in degrees or Å) between the ligand and residues.

Previous studies have demonstrated that certain BDS-related components in RR undergo near-complete degradation following processing. Subsequent to processing, the added components were selected for molecular docking with AKT1 and NOS3. The molecular docking results are presented in Figure 7A,B, with the optimal conformations illustrated in Figure 7C,D. Table 4 enumerates the binding energies between the target and each component. A bonded-energy heat map is depicted in Figure 7E. The findings indicate that melibiose, 8-epilganic acid, and mannotriose exhibited superior docking effects with potential targets, suggesting their potential significance in the intervention of BDS by RRP.

### 2.3. Behavioral Analysis of Mice

During the model development and pharmacological intervention phases, systematic behavioral observations and quantitative assessments of the mice were conducted.

#### 2.3.1. Grab Test

The grip strength of the mice was assessed using a BIO-GS3 grip tester, and the findings are presented in Table S1. The data indicated a significant reduction in grip strength among the mice in the model group. However, HRRP was found to effectively enhance grip strength and mitigate fatigue induced by BDS.

#### 2.3.2. Hair Status Score

A 3-point scale was employed to assess the surface texture, with 0 indicating smoothness, 1 indicating slight roughness, 2 representing obvious fluffiness, and 3 indicating unhairing. The results are summarized in Table S2. The score for the model group was significantly elevated (2.4 ± 0.3 compared to 0.2 ± 0.1, *p* < 0.01). The HRRP group demonstrated recovery to a score of 0.5 ± 0.2 (*p* < 0.01), which was significantly superior to the HRR group, which scored 1.6 ± 0.3.

#### 2.3.3. Dynamic Changes of Body Weight

Dynamic alterations in body weight were assessed by measuring the body weight of each group of mice on days 0 and 7 after administration. The findings are shown in Table S3. The results indicated that the body weight of mice in the model group was significantly reduced, whereas that of mice in the other administration groups, with the exception of LRR, was significantly increased.

The effect of RRP on BDS was observed through behavioral changes in mice. Following BDS induction, the mice in the model group exhibited fatigue, drowsiness, and decreased appetite, accompanied by weight loss, hair thinning, ear pallor, and diminished tail coloration. These symptoms are consistent with the TCM descriptions of BDS. In contrast, compared with the BDS-induced mice, the control subjects treated with RRP and BDS-induced mice demonstrated increased mental activity, improved appetite, weight gain, robust hair growth, and enhanced coloration of the ears and tail.

### 2.4. Routine Blood Testing

**Figure 8:**
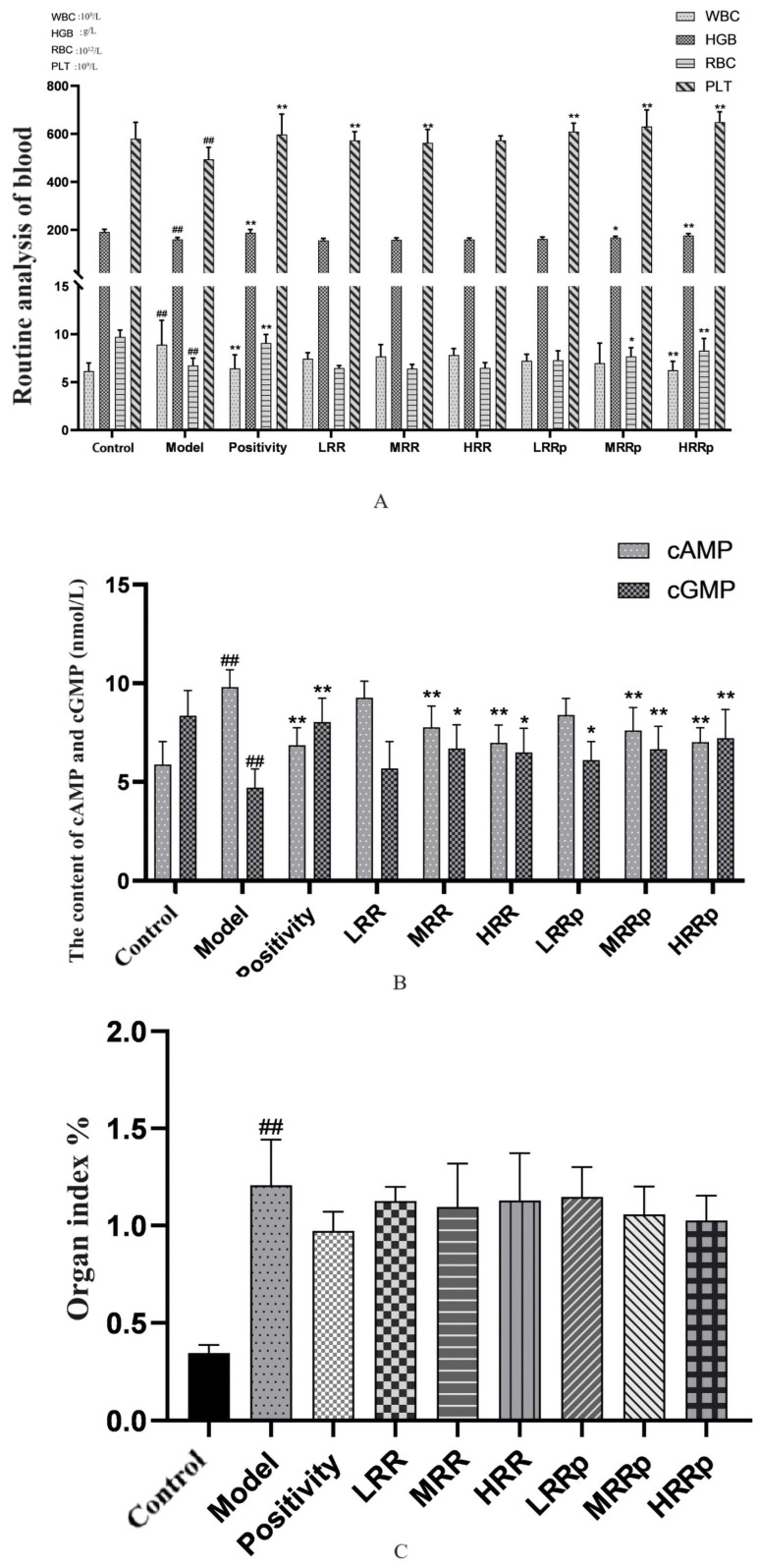
(**A**) Mouse blood routine results. (**B**) The content of cAMP and cGMP in mouse plasma. (**C**) Organ index of mouse spleen. Compared to the control group, the model group had *p* < 0.01; indicated by ^##^. Compared to the model group, the remaining dosing groups had *p* < 0.05, indicated by *, *p* < 0.01; indicated by **. Control (normal), Model (disease model), Positivity (positive control), LRR/MRR/HRR (Low, Medium, and High-Dose Rehmanniae Radix Group), LRRp/MRRp/HRRp (Low, Medium, and High-Dose Rehmanniae Radix Praeparata Group).

After 7 days of treatment, RBC, WBC, HGB, and PLT levels in the peripheral blood of the mice were measured (Figure 8A). Compared with the control group, the WBC count of the model group increased significantly (*p* < 0.01), while the HGB, RBC, and PLT contents decreased significantly (*p* < 0.01). In the positive control group, the WBC count decreased significantly (*p* < 0.01), whereas the HGB, RBC, and PLT counts increased significantly (*p* < 0.01). PLTs in the LRR, MRR, and LRRP groups were significantly elevated (*p* < 0.01). The HGB and RBC contents in the MRRP group significantly increased (*p* < 0.05), and the PLT content significantly increased (*p* < 0.01). The WBC count in the HRRP group decreased significantly (*p* < 0.01), while the HGB, RBC, and PLT levels increased significantly (*p* < 0.01). These results indicate that RRP can effectively ameliorate the BDS. The F value associated with the blood routine analysis is presented in Table S4.

After seven days of treatment, plasma cAMP and cGMP levels were measured (Figure 8B). Compared with the control group, the cAMP content in the model group increased significantly (*p* < 0.01), and the cGMP content decreased significantly (*p* < 0.01). Compared with those in the model group, the cAMP (*p* < 0.01) and cGMP (*p* < 0.01) levels in the positive control and HRRP groups were significantly lower. These findings suggest that RRP can effectively improve the energy metabolism in BDS mice. The F values corresponding to the analysis of cAMP and cGMP content are presented in Table S5.

### 2.5. Organ Index

The organ index results are presented in Figure 8C. The spleen organ index of the model group was significantly higher than that of the control group (*p* < 0.01). In comparison to the model group, the indices of positivity in the MRRP and HRRP groups were reduced. However, this difference did not reach statistical significance. The findings indicate that RRP mitigated splenomegaly induced by BDS. Following the conclusion of the experiment, spleen samples from each group of mice were collected, fixed in 4% paraformaldehyde for 72 h, dehydrated using a gradient ethanol series, and embedded in paraffin. The spleens were serially sectioned at a thickness of 5 μm along their long axis, with the spleen hilum serving as the anatomical landmark. The central region containing the largest cross-section was selected for H&E staining. Morphometric analysis was conducted utilizing ImageJ software. The pathological section of the mouse spleen is depicted in Figure S1, while the corresponding cross-sectional area results are presented in Figure S2. The findings indicated a significant increase in spleen size in the model group, and the splenomegaly in the mice was ameliorated to varying extents across each administration group, although no significant differences were observed.

### 2.6. Bone Marrow, Spleen, and Abdominal Aortic Vascular H&E Staining Observation

**Figure 9:**
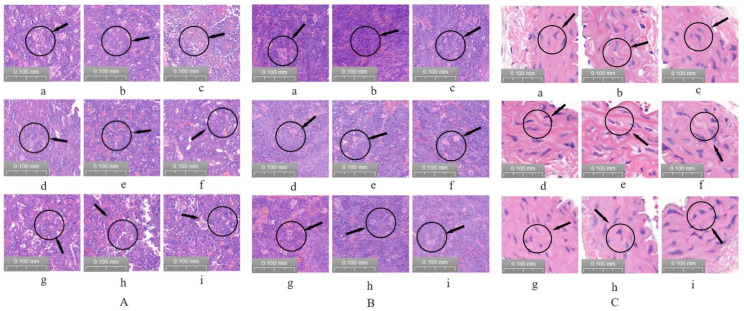
Pathological section analysis ((**A**) pathological bone marrow section, (**B**) pathological spleen section, and (**C**) pathological abdominal aorta section). Among them, a stands for the control group, b stands for the model group, c stands for the positivity group, d stands for the LRR group, e stands for the MRR group, f stands for the HRR group, g stands for the LRRP group, h stands for the MRRP group, and i stands for the HRRP group. Control (normal), Model (disease model), Positivity (positive control), LRR/MRR/HRR (Low, Medium, and High-Dose Rehmanniae Radix Group), LRRp/MRRp/HRRp (Low, Medium, and High-Dose Rehmanniae Radix Praeparata Group).

Hematoxylin and eosin (H&E)-stained tissue sections of mouse bone marrow were observed by light microscopy. The results showed that the mice in the control group had active bone marrow hyperplasia, dense nucleated cells, normal proportions of each lineage, normal morphology of mature red blood cells, more common megakaryocytes, more platelets, and a distribution in clusters. Compared with the control group, myelodysplasia, general failure, consistent size of mature red blood cells, thrombocytopenia, and megakaryocytes were rare in the model group. Compared with the model group, there were no significant changes in the LRR, MRR, or HRR groups. The positive group and the LRRP, MRRP, and HRRP groups were densely packed with nucleated cells. The proportion of each line was normal, the morphology of mature red blood cells was normal, megakaryocytes were more common, platelets were more common, and they were distributed in clusters (Figure 9A).

H&E-stained tissue sections of the mouse spleen were observed using light microscopy. The splenic lymph nodes of the white group mice had an elliptical and regular shape, and the red and white pulps were clearly demarcated. Compared with the control group, the model group mice had irregular splenic lymph node morphology, unclear demarcation of the red pulp and white pulp, and focal hyperplasia. Compared with those in the model group, there were no significant changes in the LRR, MRR, or HRR group. The splenic lymph nodes of mice in the positive control group and the LRRP, MRRP, and HRRP groups had elliptical and regular shapes, and the red and white piths were clearly demarcated (Figure 9B).

H&E-stained tissue sections of the mouse abdominal aortic vasculature were examined using light microscopy. The vascular walls of the abdominal aortas in the control group exhibited distinct layering, with a smooth and intact inner membrane. Endothelial cells were observed to be uniformly arranged, and no foam cell aggregation or collagen fiber hyperplasia was detected beneath the endothelium. The smooth muscle cells of the middle membrane were uniformly arranged without atrophy or hyperplasia, and the outer membrane morphology remained normal. In contrast, the abdominal aortas of mice in the model group demonstrated indistinct vascular wall stratification, with irregular thickening of the inner membrane, endothelial cell detachment, smooth muscle cell hyperplasia beneath the endothelium, and disorganized arrangement of smooth muscle cells in the middle membrane. The positive control group exhibited generally distinct vascular walls in the abdominal aortas, with uniformly arranged endothelial cells. No significant foam cell aggregation or collagen fiber hyperplasia was observed beneath the endothelium. Smooth muscle cells in the middle membrane were uniformly arranged without atrophy or hyperplasia, and the outer membrane morphology remained normal. No significant alterations were observed in the LRR, MRR, or HRR groups. At the vascular wall level, the LRRP, MRRP, and HRRP groups demonstrated slight thickening of the inner membrane, with scattered lipid deposition observed beneath the endothelium. Smooth muscle cell proliferation was not pronounced, and smooth muscle cells in the middle membrane were uniformly arranged (Figure 9C). ImageJ was used to quantify the proportion of bone marrow megakaryocytes within the bone marrow, spleen lymphocytes within the spleen, and arterial endothelial cells within the arteries. The results are shown in Figure S3. These results indicated that RRP could ameliorate these lesions.

RRPs have demonstrated efficacy in ameliorating the incidence of BDS lesions in the bone marrow, spleen, and abdominal aorta.

### 2.7. Results of Immunohistochemical Analysis of the Spleen and Abdominal Aorta

**Figure 10:**
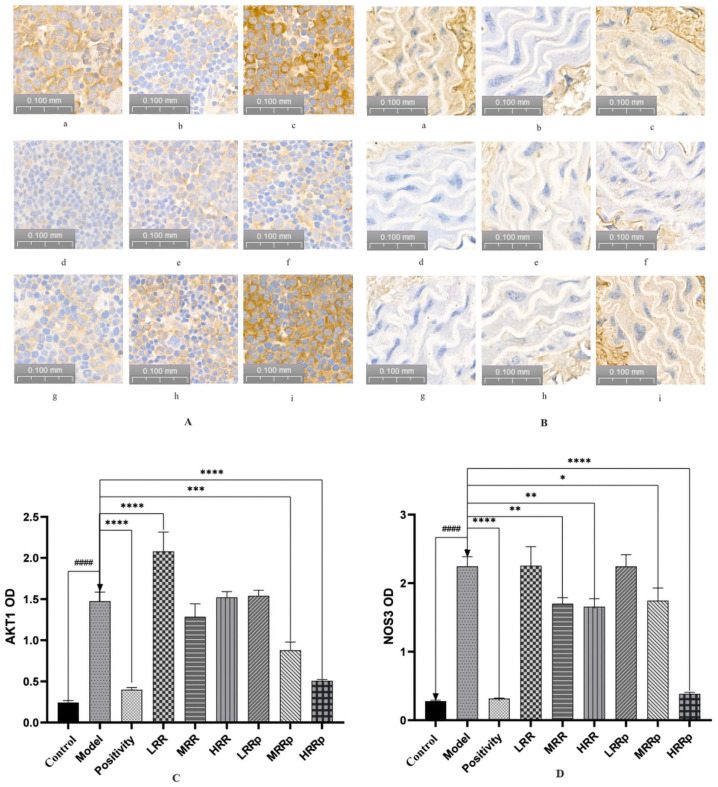
Immunohistochemical analysis. (**A**) AKT1 expression in the spleen, (**B**) NOS3 expression in the abdominal aorta, (**C**) AKT1 grayscale value analysis, (**D**) NOS3 grayscale value analysis. where a represents the control group, b represents the model group, c represents the positivity group, d represents the LRR group, e represents the MRR group, f represents the HRR group, g represents the LRRP group, h represents the MRRP group, and i represents the HRRP group. Control (normal), Model (disease model), Positivity (positive control), LRR/MRR/HRR (Low, Medium, and High-Dose Rehmanniae Radix Group), LRRp/MRRp/HRRp (Low, Medium, and High-Dose Rehmanniae Radix Praeparata Group). Compared to the control group, the model group had *p* < 0.0001; and indicated by ^####^. Compared to the model group, the remaining dosing groups had *p* < 0.05, indicated by *, *p* < 0.01; indicated by **, *p* < 0.001; indicated by ***, *p* < 0.0001; and indicated by ****.

The results of the immunohistochemical analysis are presented in Figure 10A,B. In order to reduce errors and ensure the accuracy of data, OD value correction was used. The OD value is negatively correlated with the gray value (protein expression intensity), and the correlation diagram is shown below (Figure S4). Therefore, the OD value map of IHC is opposite to the trend of Western blot protein below. But the corresponding protein trends are the same. (Figure 10C,D). RPR effectively enhanced AKT1 and NOS3 expression in the BDS group. In comparison to the control group, AKT1 and NOS3 expression was significantly reduced in the model group (*p* < 0.01). Relative to the model group, the expression levels of AKT1 and NOS3 were significantly elevated in the positive control group. The expression of AKT1 in the RR group was significantly diminished (*p* < 0.01), whereas it was significantly increased in the MRRP and HRRP groups (*p* < 0.01). NOS3 expression was significantly elevated in the MRR, HRR, and MRRP groups. The expression of NOS3 in the MRRP group exhibited significant improvement (*p* < 0.05). These findings suggest that RRP effectively enhanced the expression of AKT1 and NOS3 in BDS.

### 2.8. Expression of mRNAs in Tissues with AKT1 and NOS3

**Figure 11:**
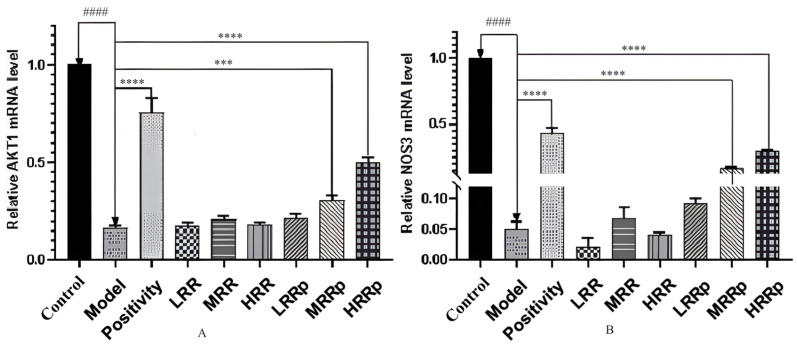
Results of RT–PCR analysis. (**A**) Expression of the AKT1 mRNA in the spleen. (**B**) Expression of the NOS3 mRNA in the kidney. Control (normal), Model (disease model), Positivity (positive control), LRR/MRR/HRR (Low, Medium, and High-Dose Rehmanniae Radix Group), LRRp/MRRp/HRRp (Low, Medium, and High-Dose Rehmanniae Radix Praeparata Group). Compared to the control group, the model group had *p* < 0.0001; and indicated by ^####^. Compared to the model group, the remaining dosing groups had *p* < 0.001; indicated by ***, *p* < 0.0001; and indicated by ****.

The primer sequences for AKT1 and NOS3 are presented in Table 2. The mRNA expression of AKT1 in the mouse spleen and NOS3 in the mouse kidney were quantified. The mRNA expression levels of AKT1 and NOS3 were significantly lower in the model group compared to the control group (*p* < 0.01). In comparison to the model group, the mRNA expression of AKT1 and NOS3 in the HRRP group was significantly reduced (*p* < 0.01) (Figure 11). These results suggest that RRP may potentially treat BDS by upregulating the mRNA expression of AKT1 and NOS3.

### 2.9. Expression of Proteins in Tissues with AKT1 and NOS3

**Figure 12:**
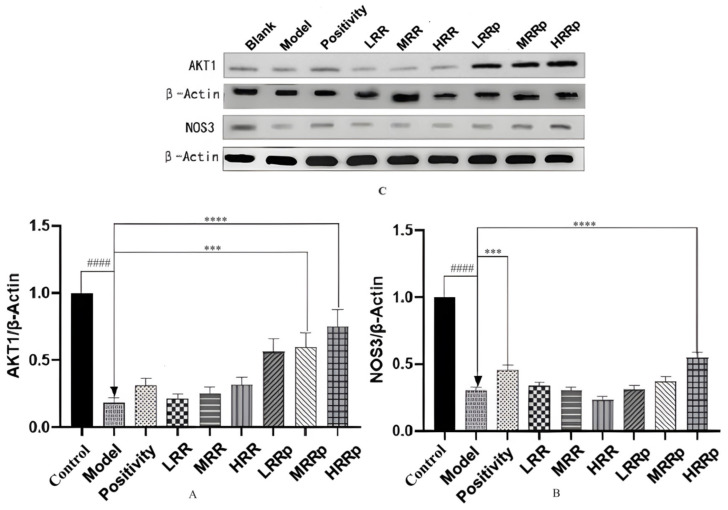
Results of WB analysis ((**A**) Protein expression of AKT1 and NOS3. (**B**) Protein expression of AKT1. (**C**) Protein expression of NOS3). Control (normal), Model (disease model), Positivity (positive control), LRR/MRR/HRR (Low, Medium, and High-Dose Rehmanniae Radix Group), LRRp/MRRp/HRRp (Low, Medium, and High-Dose Rehmanniae Radix Praeparata Group). Compared to the control group, the model group had *p* < 0.0001; and indicated by ^####^. Compared to the model group, the remaining dosing groups had *p* < 0.001; indicated by ***, *p* < 0.0001; and indicated by ****.

The protein expression of AKT1 in the mouse spleen and NOS3 in the mouse kidney were quantified. The protein expression of AKT1 and NOS3 is illustrated in Figure 12A. Compared to the control group, the protein expression levels of AKT1 and NOS3 were significantly lower in the model group (*p* < 0.01). Relative to the model group, the protein expression of AKT1 in the RRP-treated group and the middle- and high-dose RRP groups was significantly reduced (*p* < 0.01). In comparison to the model group, the protein expression of NOS3 in the positive control group and the high-dose RRP group was significantly lower (*p* < 0.01) (Figure 12B,C). These findings suggest that RRP may potentially treat BDS by upregulating the expression of the AKT1 and NOS3 proteins.

## 3. Discussion

The primary chemical constituents of *Rehmannia glutinosa* are iridoid glycosides, phenylethanoid glycosides, amino acids, and sugars. During processing, the majority of iridoid glycosides undergo thermal decomposition, resulting in the cleavage of glycosidic bonds and the formation of glucose; the corresponding aglycones, such as catalpol, are almost entirely decomposed post-processing [10]. Polysaccharides (*Rehmannia glutinosa* polysaccharides a, b, SRP I, SRP II [11], etc.), oligosaccharides, mannose, raffinose, and other components of *Rehmannia glutinosa* readily decompose, producing a substantial quantity of monosaccharides. Furthermore, the Maillard reaction generates 5-HMF and melanin-like substances during the steaming process of *Rehmannia glutinosa*. Alterations in these chemical components may be the fundamental cause of the differing effects observed between raw and processed *Rehmannia glutinosa*. However, current research on the differential components of raw and processed *Rehmannia glutinosa* is limited, and the material basis of the various treatments used to prepare *Rehmannia glutinosa* remains unclear. Consequently, this study employed UPLC-Q-TOF-MS to qualitatively analyze the chemical constituents of RR and RRP. In conjunction with partial least squares analysis, the identified compounds were screened to determine the differential components of RR and RRP, as well as changes in other components. The objective of this study was to investigate the material basis of different treatments for *Rehmannia glutinosa* and to provide theoretical support for related research on this material. A total of 59 components exhibiting altered expression post-processing were identified for positive ions, while 39 components with changed expression post-processing were identified for negative ions. These compounds encompass iridoids, phenylethanoid glycosides, carbohydrates, benzaldehydes, phenylpropanoid glycosides, organic acids, dihydroflavonoids, 5-hydroxymethylfurfural (HMF), and their derivatives. The mechanism underlying the changes in chemical composition following the processing of *Rehmannia glutinosa* may involve the thermal decomposition of iridoids, phenylethanoid glycosides, sugars, and other compounds, resulting in the generation of new similar compounds or other compounds.

According to scientific and medical perspectives, the pathogenesis of blood deficiency syndrome (BDS) may involve impaired blood production, metabolic imbalances, circulatory dysfunction, and organ system disorders. Consequently, Chinese herbal medicines that can enhance blood production, improve metabolic function, and support bone marrow health are frequently utilized in clinical practice to treat BDS and anemia. Radix Rehmanniae Praeparata (RRP) is commonly employed in bulk Chinese medicine in clinical practice. It has been used for over 1000 years to promote blood production and improve metabolic health; however, the precise mechanism of its hematopoiesis-enhancing effect remains unclear. Therefore, this study investigated the mechanism of action of RRP in the treatment of BDS. As the cyclic adenosine monophosphate (cAMP) concentration increased, the cyclic guanosine monophosphate (cGMP) concentration decreased. Peripheral red blood cell (RBC), white blood cell (WBC), hemoglobin (HGB), and platelet (PLT) counts were significantly reduced, a typical clinical manifestation of BDS and anemia [12]. Another indication of anemia is splenomegaly, which is one of the most common and earliest pathological manifestations of various types of anemia [13]. It is generally accepted that hematopoietic dysfunction is associated with the discharge of bone marrow cells from the bone marrow into the blood, resulting in accumulation in the spleen [14].

The accumulation of RBCs in the enlarged spleen can exacerbate anemia. Consequently, the reduction in circulating RBCs and anemia is correlated with the degree of spleen enlargement. This experimental study revealed that the cAMP concentration increased as the cGMP concentration decreased. HGB, RBC, and PLT content increased significantly. Additionally, organ index experiments demonstrated that RRP inhibited splenomegaly in BDS mice. These findings suggest that RRP could be efficacious in treating BDS.

Although this study elucidated the regulatory effects of RRP on peripheral hematopoietic function and the PI3K-AKT signaling pathway in blood deficiency models, technical limitations—including challenges in obtaining bone marrow samples and isolating hematopoietic stem cells (HSCs)—precluded direct validation of its mechanisms within the bone marrow microenvironment. While alterations in peripheral blood indices (e.g., RBC, HGB) and splenic parameters may indirectly reflect improved hematopoietic function [15], future investigations should prioritize the following validations: ① Single-cell RNA sequencing of bone marrow to delineate HSC differentiation trajectories under RRP intervention; ② Quantification of PI3K-AKT pathway core proteins (e.g., AKT1 and p-AKT) and downstream hematopoietic factors (e.g., EPO and SCF) in bone marrow stroma; ③ In vitro co-culture experiments with bone marrow-derived cells to identify RRP’s direct cellular targets. These limitations do not compromise the reliability of the current findings but highlight the need for spatial multi-omics approaches to unravel tissue-specific pharmacological actions of RRP.

The dysregulation of cyclic nucleotides (cAMP and cGMP) may critically contribute to BDS progression by disrupting platelet and erythrocyte differentiation. cAMP enhances megakaryopoiesis through PKA-mediated activation of RUNX1 and GATA1 [16], while its depletion in BDS likely exacerbates thrombocytopenia. Conversely, cGMP promotes erythropoiesis via PKG-dependent STAT5 phosphorylation [17], but excessive nitric oxide (NO) from NOS3 upregulation paradoxically depletes cGMP through oxidative inactivation of soluble guanylate cyclase (sGC) [18], thereby impairing erythrocyte maturation. Notably, network pharmacology predicts that RRP’s components (e.g., baicalin) target phosphodiesterases (PDE4/5), which degrade cAMP/cGMP, suggesting that RRP may restore cyclic nucleotide homeostasis to alleviate BDS. This aligns with our findings that RRP rescues AKT1-NOS3 imbalance, as AKT1 potentiates NO synthesis (via NOS3 activation), while NO feedback inhibits AKT1 through S-nitrosylation, forming a regulatory loop that intersects with cAMP/cGMP signaling. Future studies should directly measure cyclic nucleotide levels in BDS models to validate this mechanistic axis.

This study employed a network pharmacology approach to elucidate the therapeutic mechanisms and key targets of RRP in treating bleeding disorder syndrome (BDS). Analyses revealed that RRP may exert its effects through platelet regulation. Platelets, anucleate cellular fragments derived from bone marrow megakaryocytes, constitute only 0.05% of nucleated bone marrow cells but play a central role in hemostasis and blood circulation. Histopathological sections demonstrated that RRP significantly alleviated BDS-induced bone marrow damage, suggesting its potential to improve hematopoietic function by protecting megakaryocyte differentiation or modulating platelet activity. KEGG pathway enrichment analysis further indicated that RRP’s active components likely regulate BDS progression via the PI3K/AKT/mTOR signaling pathway, with AKT1 and NOS3 identified as pivotal targets. Molecular docking confirmed strong binding affinities between RRP’s bioactive compounds and AKT1/NOS3 (binding energy < −7.0 kcal/mol), providing structural evidence for their targeted modulation.

AKT1, a core mediator of the PI3K/AKT/mTOR pathway, contributes to BDS pathogenesis by balancing pro-/anti-inflammatory responses and metabolic reprogramming. On one hand, AKT1 phosphorylation (Ser473) promotes IL-6 and TNF-α release via NF-κB activation while suppressing FOXO1-mediated IL-10 expression [19], fostering a pro-inflammatory microenvironment. Concurrently, AKT1 upregulates glycolytic enzymes (e.g., HK2 and LDHA), inducing a Warburg effect in macrophages to sustain inflammation energetically [20]. Complementarily, NOS3 upregulation highlights the dual role of nitric oxide (NO): early-stage NO production via cGMP-dependent vasodilation improves tissue perfusion, but chronic NO elevation induces mitochondrial dysfunction and oxidative stress [21]. Furthermore, NO exacerbates hematopoietic impairment by forming methemoglobin (reducing oxygen transport) and inhibiting STAT5 phosphorylation (blocking erythrocyte differentiation) [22]. Future studies should employ cell-specific knockout models (e.g., macrophage AKT1 KO or endothelial NOS3 KO) to dissect spatiotemporal regulatory mechanisms, enabling precise therapeutic targeting.

From a pharmacodynamic perspective, in addition to regulating peripheral blood and the levels of cAMP and cGMP, RRP can also modulate the expression of AKT1 and NOS3 during the treatment of BDS. Western blot analysis revealed that the protein expression levels of AKT1 and NOS3 were significantly upregulated in the HRRP group. RT-qPCR results demonstrated that the protein expression levels of AKT1 and NOS3 were significantly elevated. The variation trends of the two results were consistent, corroborating the reliability of the network pharmacology predictions. Combined with network pharmacology and pharmacodynamics analyses, the platelet activation pathway may be associated with BDS, and the PI3K-AKT signaling pathway may be the key pathway of RRP in the treatment of BDS. This may represent a potential mechanism for RRP in the treatment of BDS. In addition, pathways such as TLR4/MyD88 may also be involved in the progress of BDS, which needs to be further studied in future experiments, and the targets affecting the production of cAMP and cGMP need to be further identified and tested.

However, the mechanism of RRP in the treatment of BDS necessitates further investigation, such as metabolomic research. These aspects will be examined in subsequent studies. This research provides comprehensive theoretical support for the clinical application of RRP.

## 4. Materials and Methods

### 4.1. Instruments and Materials

An ACQUITY UPLC I-CLASS ultrahigh-performance liquid chromatography-tandem Xevo G2-XS Q-TOF mass spectrometer (Waters Corporation, Milford, MA, USA), kQ-250DB CNC ultrasonic cleaner (Kunshan Ultrasonic Instrument Co., Ltd., Kunshan, Jiangsu, China), 3K15 desktop high-speed refrigerated centrifuge (SIGMA Laborzentrifugen GmbH, Osterode am Harz, Germany), Milli-Q Integral pure water/ultrapure water integrated system (Millipore, Burlington, MA, USA), fA1004B electronic balance (Shanghai Precision Scientific Instrument Co., Ltd., Shanghai, China), mS105DU analytical balance (METTLER TOLEDO, Greifensee, Switzerland), mass spectrometry-grade formic acid (Merck, Darmstadt, Germany), chromatographic grade methanol and mass spectrometry grade acetonitrile (Fisher Chemical Company, Pittsburgh, PA, USA), and tOF G2-S Sample Kit-1 (lot number W21111602, Waters, Milford, MA, USA) were used. Rehmanniae Radix Praeparata and Rehmanniae Radix were purchased from the Sichuan New Lotus Traditional Chinese Medicine Tablet Center (lot numbers: 2209026, 2205018), AKT1 (#2938S, Cell Signaling Technology, Danvers, MA, USA), NOS3 (ab199956, Abcam, Cambridge, UK), β-actin (#4970, Cell Signaling Technology, Danvers, MA, USA), cyclophosphamide (Ltd. H32020857, Jiangsu Hengrui Pharmaceutical Co., Lianyungang, Jiangsu, China), and acetylphenylhydrazine (Ltd. 1099092, Shanghai Aladdin Biochemical Technology Co., Shanghai, China). Compound Ejiao pulp (Ltd. Z20083345, Donge Ejiao Co., Dong’e, Shandong, China).

### 4.2. Preparation of Test Solution

Approximately 0.2 g of sample powder was weighed, precisely weighed, placed in a conical flask with a stopper, mixed with 20 mL of 50% methanol, weighed, sonicated for 30 min, cooled, weighed, and supplemented with methanol. The lost mass was shaken well, filtered, and the filtrate was removed.

### 4.3. Detection Conditions

The chromatographic conditions were as follows: Waters Acquity UPLC BEH Amide C18 column (2.1 mm × 100 mm, 1.7 μm), equipped with a Waters Acquity UPLC BEH Amide C18 V precolumn (2.1 mm × 5 mm, 1.7 μm); mobile phase: A (0.1% formic acid water): B (0.1% formic acid acetonitrile); gradient elution: 0~1 min, 5~5% A; 1–15 min, 5–35% A; 15–25 min, 35–40% A; 25–27 min, 40–40% A; 27–27.01 min, 40–5% A; 27.01–30 min, 5–5% A. The injection volume was 2 μL, and the flow rate was 0.40 mL/min.

### 4.4. Mass Spectrometry Conditions

The following parameters were used: electrospray ionization (ESI) source, mass spectrometry detection and analysis in positive and negative ion modes, data acquisition as a continuum, scanning range of *m*/*z* 50–1000, MSE scanning mode detection, low-energy scanning (function 1), transmission collision energy of 0 eV, and high-energy scanning (function 2), transmission collision energy of 20–30 eV. Leucine-enkephalin (*m*/*z* 554.2615 [M − H]^−^, *m*/*z* 556.2771 [M + H]^+^) was used for calibration. Capillary voltage: positive ion: 2.0 kV; negative ion: 2.5 kV; sampling cone voltage: 40 V; ion source compensation voltage (source offset): 80 V; ion source temperature: 100 °C; desolvation temperature: 400 °C; cone gas flow: 100 L · h^−1^; desolvation gas flow: 800 L · h^−1^.

Establishment of chemical constituents database of RR: By consulting the literature on the compounds contained in RR and its plants of the same family and genus in PubMed, ChemSpider, China National Knowledge Infrastructure (CNKI), Wanfang, Weipu, and other databases and searching for the chemical structure of the relevant compounds on the PubChem website, ChemDraw software 15.1 was used to construct the compound structure, which was saved as a mol-format file, and an Excel table was established (including the compound name and corresponding information mol structural file) and imported into UNIFI 1.9.4 software (Waters, Milford, MA, USA). A database of the chemical constituents of *Rehmannia glutinosa* pills was generated.

Data processing of chemical constituents: The UPLC-Q-TOF-MSE mode was used to collect data. First, the self-built *Rehmannia glutinosa* chemical composition database in UNIFI software was used to supplement the original natural product chemical composition database, and then a filtering screening method was established: the retention time range was 0.5–20 min, the response intensity threshold was ≥5000, and the mass error was 10 ppm. Finally, qualitative identification of chemical components was carried out by combining the cracking law of compounds, retention time, and distribution in samples (excluding the chemical components contained in control samples and unstable distribution in QC). SIMCA-P 14.1 software was used for PCA and OPLS-DA to determine the differences in chemical components between RR and RRP.

### 4.5. Network Analysis

Network pharmacological analysis was performed on the screened differential components. The targets of these components were screened via the Traditional Chinese Medicine Systems Pharmacology Database and Analysis Platform (TCMSP), Swiss Target Prediction, TargetNet [23], and PharmMapper. A total of 369 targets were screened. In addition, GeneCards and Online Mendelian Inheritance in Man (OMIM) were used to screen targets related to BDS. The keyword “blood deficiency syndrome” was entered into the GeneCard database, a relevance score > 20 was selected, and a total of 701 critical targets were obtained. A Venn diagram of the two intersecting 82 genes was generated.

To analyze the common targets of RRP pharmacodynamic effects and BDS, the Metascape server [24] was used for Kyoto Encyclopedia of Genes and Genomes (KEGG) and Gene Ontology (GO) enrichment analyses.

The PPI network was constructed with the STRING database. The constructed network was then analyzed by software. All networks were constructed with Cytoscape 3.8.0 [25].

The pathways screened by pathway enrichment analysis were compared and combined with the key targets screened by the PPI network. As a result, the key target in the selected pathway was identified, which may be a key target for the treatment of BDS by RRP.

The 2D structural information of different components was obtained from the TCMSP database and the PubChem database. This 2D information was imported into ChemDraw 3D for energy minimization, and the corresponding 3D structure was generated. Moreover, the 3D structure of the target protein was downloaded via the PDB database [https://www.rcsb.org/ (URL: 17 March 2023)]. OpenBabel 3.1.1 software and AutodockTool 4.2 software were used to hydrogenate and discharge small molecules and target proteins, respectively. Docking active sites were found with the help of the PyMOL 2.5 software plugin. Using AutoDock Vina 1.2.2 software, the treated components were molecularly docked with the target protein. Finally, the docking results were visualized by PyMOL 2.5 software.

### 4.6. Animals and Ethics Statement

Clinical trial number: not applicable. This study employed 72 healthy male KM mice with a weight range of 18–22 g (batch number SCXK (Liao) 2020–0001) obtained from Liaoning Changsheng Biotechnology Co., Ltd. (Shenyang, China) The animal experiment received approval from the Ethics Committee of Liaoning University of Traditional Chinese Medicine (approval number 2018YS(DW)-041-01). The animals were maintained in an environment with a temperature of 25 °C and relative humidity of 40–60%. The mice were randomly allocated into five groups, each comprising 8 mice: the control group, model group, positivity group, low-dose Rehmanniae Radix group (LRR), middle-dose Rehmanniae Radix group (MRR), high-dose Rehmanniae Radix group (HRR), low-dose Rehmanniae Radix Praeparata group (LRRP), middle-dose Rehmanniae Radix Praeparata group (MRRP), and high-dose Rehmanniae Radix Praeparata group (HRRP).

### 4.7. Establishment of the Blood Deficiency Syndrome Model

Acetylphenylhydrazine (APH) (20 mg/kg, 10 mg/kg) was administered subcutaneously on the first and fourth days. On the fourth day, 2 h after subcutaneous administration of APH, cyclophosphamide (CP) (20 mg/kg) was administered intraperitoneally. CP was administered for 4 consecutive days thereafter. The control group received an equivalent volume of saline via the same route [26]. On the seventh day, the percentages of red blood cells (RBCs), white blood cells (WBCs), hemoglobin (HGB), and platelets (PLTs) in the peripheral blood were quantified. According to the *Pharmacopoeia of the People’s Republic of China 2020 Edition* and the body surface area conversion formula, the equivalent dose for mice was 0.91–3.64 g/kg. From the first day, the treatment groups received RR or RRP (1 g/kg, 2 g/kg, or 4 g/kg) via oral gavage. The control group and model group received an equivalent volume of saline via oral gavage for 10 days.

### 4.8. Biochemical Index Detection of BDS Mice

#### 4.8.1. Routine Blood Test

Subsequent to the final administration, blood was extracted from the abdominal aorta and collected in a sterile vacuum blood collection tube containing ethylenediaminetetraacetic acid (EDTA). The blood was subsequently analyzed using an Animal-6008 automatic hematology analyzer (Shenzhen Mindray Bio-Medical Electronics Co., Ltd., Shenzhen, Guangdong, China) to quantify RBC, WBC, HGB, and PLT.

#### 4.8.2. Determination of cAMP and cGMP Content in Plasma

Two days prior to the termination of administration, venous blood samples were obtained from each group of mice using sterile vacuum blood collection tubes containing heparin sodium. Enzyme-linked immunosorbent assay (ELISA) was employed to quantify plasma cAMP and cGMP levels.

#### 4.8.3. Pathological Observation

The femurs, spleens, and livers were fixed with 4% paraformaldehyde for a minimum of 48 h, dehydrated using a graded alcohol series, embedded in paraffin, and sectioned. Following desiccation, xylene deparaffinization, washing, and hematoxylin and eosin (H&E) staining, the morphology of the femoral, splenic, and hepatic tissues from each experimental group was examined under light microscopy.

#### 4.8.4. Immunohistochemical Analysis

The paraffin-embedded kidney sections were deparaffinized and hydrated. Following antigen retrieval, the sections were incubated with primary antibodies against AKT1 (#K101311P, Solarbio Life Sciences Co., Ltd., Beijing, China) and NOS3 (#K112744P; Solarbio Life Sciences Co., Ltd., Beijing, China) overnight at 4°C. Subsequently, the sections were incubated with a secondary antibody (#ab7090; Abcam) for 2 h, followed by counterstaining with hematoxylin. The positively stained sections were observed and analyzed using a BX51 microscope. The immunohistochemistry results were analyzed using ImageJ V1.8.0.112 software.

### 4.9. RT-qPCR Analysis

The frozen kidney and spleen were extracted from the −80 °C ultralow temperature freezer and homogenized utilizing liquid nitrogen. Total RNA was isolated according to the TRIzol kit protocol, and cDNA was synthesized via reverse transcription, with Gapdh serving as the internal reference. Quantitative PCR primer sequences were designed utilizing Primer Premier 6.0 and Beacon Designer 7.8 software. The detailed sequences are presented in Table 5. The volume of the RT-qPCR amplification system was 20 μL, comprising 1 μL of cDNA, 0.5 μL each of upstream and downstream primers, 10.0 μL of Power SYBR Green Master Mix (AG11718, Accurate Biotechnology Ltd., Changsha, China), and sterile distilled water to a final volume of 20 μL. The reaction conditions were as follows: predenaturation at 95 °C for 1 min; amplification at 95 °C for 15 s and 63 °C for 25 s, for 40 cycles; melting point curve analysis at 55–95 °C; and amplification on a StepOne™ PCR instrument. The 2^−ΔΔCT^ method was employed to compare the relative expression levels of AKT1 and NOS3 in each group.

### 4.10. Western Blot Analysis

The frozen kidney and spleen were extracted from the −80 °C ultralow temperature freezer and pulverized with liquid nitrogen. An RNAex (AG21102, Accurate Biotechnology Ltd.) lysate kit was utilized for protein extraction. The sample protein concentration was quantified using a BSA (Cat# PC0020, Solarbio) protein kit. Subsequently, the samples were diluted to the lowest concentration with 5× loading buffer and PBS. The proteins were separated via 10% SDS polyacrylamide gel electrophoresis and transferred to PVDF membranes. The membrane was blocked with 5% skim milk at room temperature for 1 h, followed by the addition of antibodies against AKT1, NOS3 (all 1:1000), and β-Actin (all 1:2000). The membranes were incubated overnight at 4 °C and washed with Tris-buffered saline containing Tween 20 (TBST) three times for 10 min each. The membrane was then immersed in horseradish peroxidase-labeled secondary antibody (1:1000), diluted in 2% skim milk, incubated at room temperature for 1 h, and washed with TBST 3 times for 10 min each. The enhanced chemiluminescence (ECL) method was employed to acquire the signal on the Image Quant LAS 500 imaging equipment.

## Figures and Tables

**Table 1:** The main chemical compositions of *Rehmannia glutinosa*.

Serial Number	Name of Compound	Chemical Formula	Molecular Weight
1	Catalpol	C_15_H_22_O_10_	362.33
2	Melittoside	C_21_H_32_O_15_	524.47
3	Geniposide	C_17_H_24_O_10_	388.37
4	Leonuride	C_15_H_24_O_9_	348.35
5	Rehmannioside A	C_21_H_32_O_15_	524.47
6	Rehmannioside B	C_21_H_32_O_15_	524.47
7	Verbascoside	C_29_H_36_O_15_	624.59
8	Isoacteoside	C_29_H_36_O_15_	624.59
9	Echinacoside	C_35_H_46_O_20_	786.73
10	Glucose	C_6_H_12_O_6_	180.16
11	Melibiose	C_12_H_22_O_11_	342.30
12	Raffinose	C_11_H_20_O_10_	312.27
13	Stachyose	C_24_H_50_O_25_	738.64
14	Manninotriose	C_18_H_32_O_16_	504.44

**Table 2:** Related information of chemical constituents in RR and RRP in positive ion mode.

Component Name	Observed *m*/*z*	Mass Error (mDa)	Observed RT (min)	Adducts	Differentiating Ingredients	Content
Rehmaionoside B	391.2326	0	1.27	+H	Yes	+
Isoeugenol methyl ester	179.1065	−0.1	1.31	+H	Yes	−
Dihydroxy-β-ionone	249.1482	2	1.32	+Na	No	
Eugenol	165.0923	1.3	1.32	+H	Yes	−
Stigmasterol	413.3789	1.1	1.32	+H	Yes	+
1,5-bis(5-methoxymethyl)furan-2-yl-yenta-1,4-dien-3-one	325.1058	1.1	1.38	+Na	No	
Jinshengcaoflavin	301.0731	2.5	1.57	+H	No	
5-hydroxymethyl furaldehyde	127.0384	−0.6	1.64	+H	Yes	+
Rehmannia new terpenoid K	712.3136	−1.2	1.67	+H	No	
5-Hydroxymethylfurancarboxylic acid	143.0333	−0.6	1.85	+H	Yes	−
Kojic acid	269.1759	1.1	2.04	+H	Yes	−
Nicotine	163.1223	−0.7	2.69	+H	Yes	+
Isotachioside	303.108	0.6	3.05	+H	Yes	+
Masmin	147.0903	−1.4	3.14	+H	No	
Sculponiside	713.2817	3.7	3.69	+Na	Yes	−
(2-phenylethyl-O-β-D-xylopyranosyl-(1→6)-β-D-glucopyranoside)	413.2144	−2.6	3.79	+H	No	
Nornicotine	149.107	−0.3	4.32	+H	No	
Rhodioloside	323.1105	0.4	5.07	+Na	No	
1,2,5,6-tetrahydro-1-methyl-2-oxo-4-pyridine acetic acid	170.0801	−1	5.08	+H	No	
Uracil	113.0337	−0.9	5.52	+H	No	
6-O-vanillate ajugol	521.1672	4.3	5.53	+Na	No	
Gardoside methyl ester	389.144	−0.2	5.56	+H	Yes	+
Oxyrehmaionoside B	429.2091	−0.4	5.84	+Na	Yes	−
Rehmaionoside C	411.2002	1.3	5.98	+Na	Yes	−
JionosideD	639.2329	4.5	6.09	+H	Yes	−
Nicotyrine	181.0725	−1.1	6.1	+Na	Yes	+
Myobontioside A	350.1202	−0.6	6.12	+H	Yes	+
2,4-Dimethoxy-2-methyl-6H-pyran-3-one	195.0638	1.1	6.38	+Na	Yes	+
Trihydroxy-β-ionone	265.1426	1.6	6.42	+Na	No	
Isoquinoline	130.0646	−0.5	6.42	+H	Yes	−
Rehmannia new terpenoid D	431.2279	0.3	6.63	+H	Yes	−
Verbascoside	625.2157	3	7.01	+H	Yes	−
6-O-sec-hydroxyaeginetoyl ajugol	637.2837	0.7	7.08	+Na	No	
(E)-4-(5-(hydroxymethyl)furan-2-yl)but-3-en-2-one	189.0537	1.5	7.29	+Na	No	
Mussaenosidic acid	377.1479	3.6	7.3	+H	Yes	−
Herniarin	177.0553	0.7	7.36	+H	No	
Lamiol	401.1418	−0.1	7.52	+Na	Yes	+
Adenine	136.0614	−0.3	7.59	+H	No	
Adenosine	268.1046	0.5	7.6	+H	No	
5-Deoxylamiol	363.1619	−3.1	7.85	+H	Yes	−
Myoporoside	349.1492	−0.1	7.86	+H	Yes	+
Genipin 1-O-a-L-rhamnopyranosyl(1→6)-β-D-glucopyranoside	535.2028	0.6	7.89	+H	Yes	+
Hydrocoumarin	149.0594	−0.3	8.01	+H	No	
Jionoside B1	815.3007	3.8	8.16	+H	Yes	−
Uridine	245.076	−0.8	8.17	+H	No	
Martynoside	653.2462	2.2	8.18	+H	No	−
Galactose	203.053	0.4	9.12	+Na	No	
Jionoside A1	801.2876	6.5	9.2	+H	Yes	+
Cis-asarone	231.1008	1.7	9.38	+Na	No	
Dihydrocatalpol	387.1273	1.1	9.4	+Na	Yes	−
Ethylvanillin	167.0712	0.9	9.4	+H	Yes	−
Ferulic acid	195.0662	1	9.61	+H	Yes	+
Catalpinoside	385.1111	0.6	10.09	+Na	Yes	−
Coumarin	147.0436	−0.5	12.03	+H	Yes	−
Benzoic acid	123.0442	0.2	12.04	+H	Yes	+
Rehmannioside C	533.1837	−0.3	12.17	+Na	No	
Dibutylphthalate	301.1419	0.8	12.31	+Na	Yes	+
Syringic acid	199.0596	−0.5	12.51	+H	Yes	−
Feruloyl ajugol	743.2507	−1.4	13.29	+Na	No	
7-methylcoumarin	161.0605	0.8	13.81	+H	Yes	−
Rehmannia new terpenoid J	696.2809	−2.6	13.83	+H	Yes	+
(7R,8S,7′R,8′,S′)-4,9,4′,9′-tetrahydroxy-3,3′-dimethoxy-7,7′-epoxylignan 9-O-β-D-glucopyranoside	539.2115	−0.8	14.15	+H	Yes	+
Dhydrophaseic acid 4′-O-β-D-glucopyranoside	467.1887	−0.1	14.3	+Na	Yes	+
6-O-E-caffeoyl ajugol	533.1609	−2.1	14.44	+Na	No	
Rhinanthin	347.1345	0.8	14.72	+H	Yes	−
Manninotriose	527.161	2.7	14.77	+Na	No	
Retinoid D	709.2195	3.4	14.81	+Na	No	
Sucrose	365.1065	1.1	15.02	+Na, +H	No	
Sec-hydroxyaeginetic acid	307.153	1.4	15.26	+Na	No	
Catapolgenin	201.0757	0	15.27	+H	No	
Stachyose	667.2305	1.3	15.52	+H	Yes	−
Melittin monoglucoside	363.1289	0.3	16.03	+H	Yes	−
Rehmannia new terpenoid B	323.1461	−0.4	16.3	+Na	No	
Rehmannia neoterpene E	447.2224	−0.1	16.8	+H	Yes	−
Raffinose	505.1765	0.2	17.03	+H, +Na	No	
Rehmannioside A	525.1795	−1.8	18.05	+H	No	
Rehmannia new glucoside A	495.2258	3.3	18.08	+H	Yes	+
yemuoside YM11	507.1903	4.3	18.86	+H	Yes	+
7-ethoxy-4-methylcoumarin	227.0663	−1.5	19.82	+Na	Yes	−

‘+’ indicates that the content of this component in RRP was greater than that in RR, and ‘−’ indicates that the content of this component in RR was greater than that in RRP.

**Table 3:** Related information of chemical constituents in RR and RRP in negative ion mode.

Component Name	Observed *m*/*z*	Mass Error (mDa)	Observed RT (min)	Adducts	Differentiating Ingredients	Content
Myobontioside A	433.2053	−2.6	1.27	+HCOO	Yes	−
vanillin	457.2123	4.3	1.4	+HCOO	Yes	+
Rehmannia new glucoside B	211.1332	−0.8	1.42	-H, +HCOO	Yes	+
verbascoside	455.3524	−0.6	1.42	-H	Yes	−
Dihydroxy-β-ionone	225.1483	−1.4	1.46	-H, +HCOO	No	
5-hydroxymethyl-2-furancarboxaldehyde	429.2487	−0.7	1.47	-H	Yes	+
p-methoxycinnamic acid	223.0983	0.7	1.5	+HCOO, -H	Yes	+
yemuoside YM1	209.0836	1.7	1.51	+HCOO	No	
Rehmapicrogenin	183.1013	−1.4	1.51	-H	No	
1,5-bis(5-methoxymethyl)furan-2-yl-yenta-1,4-dien-3-one	347.1164	2.8	1.54	+HCOO, -H	Yes	−
(2-phenylethyl-O-β-D-xylopyranosyl-(1→6)-β-D-glucopyranoside)	205.0498	−0.8	1.54	+HCOO	Yes	−
7-ethoxy-4-methylcoumarin	249.0777	0.9	1.55	+HCOO	Yes	+
sucrose	193.0498	−0.8	1.56	-H, +HCOO	Yes	−
ajugol	621.4376	0.4	1.57	+HCOO	Yes	−
eugenol	225.0768	0	1.59	-H	No	
Rehmannioside C	223.0606	−0.6	1.6	+HCOO, -H	Yes	−
ethylvanillin	165.0544	−1.3	1.61	-H, +HCOO	Yes	+
herniarin	221.0451	−0.5	1.62	+HCOO	Yes	+
ferulic acid	151.0388	−1.2	1.64	-H, +HCOO	Yes	+
sec-hydroxyaeginetic acid	283.1535	−1.6	1.64	-H	Yes	−
Genipin-gentiobioside	213.075	−1.8	1.67	+HCOO	Yes	−
rhinanthin	199.0601	−1.1	2.07	-H	Yes	+
succinic acid	197.0444	−1.1	2.55	-H	No	
5-Hydroxymethylfurancarboxylic acid	187.024	−0.8	2.73	+HCOO	No	
Trihydroxy-β-ionone	287.1517	1.7	2.96	+HCOO	No	
Isoscrophularigenin	215.0918	−0.7	3.13	+HCOO	Yes	+
1,2,5,6-tetrahydro-1-methyl-2-oxo-4-pyridineacetic acid	214.0711	−1	3.15	+HCOO	No	
benzoic acid	167.0341	−0.9	3.16	+HCOO	Yes	+
3-methoxy-4-hydroxybenzoic acid	213.0395	−0.9	3.21	+HCOO, -H	Yes	+
Rehmaionoside B	301.0928	−0.1	3.28	-H	Yes	−
Rehmannia new terpenoid D	429.2115	−1.5	4.25	-H, +HCOO	No	
Oxyrehmaionoside B	435.2274	3.8	4.3	+HCOO, -H	Yes	−
Rehmannia new glucosideJ	345.1552	−0.2	4.32	+HCOO	Yes	+
Melittin	697.2363	1.4	4.47	+HCOO, -H	Yes	+
6-O-E-feruloyl ajugol	569.1908	3.2	4.59	+HCOO, -H	Yes	−
8-epiloganic acid	375.1293	−0.4	4.63	-H	No	
Geniposide	345.1182	−0.9	4.68	-H	No	
6-O-vanillate ajugol	543.1729	1	4.72	+HCOO, -H	Yes	−
Tachioside	163.0237	−1.1	4.77	+HCOO	No	
Cis-rehmannioside	449.2035	0.6	4.81	+HCOO	Yes	−
geniposide	433.1354	0.2	4.81	+HCOO	Yes	+
Choulongjianoside A	433.1355	0.4	4.82	+HCOO	Yes	−
JionosideD	637.2146	0.8	4.96	-H	No	
Angeloside C	243.0613	−1	4.96	-H, +HCOO	Yes	+
(7R,8S,7′R,8′,S′)-4,9,4′,9′-tetrahydroxy-3,3′-dimethoxy-7,7′-epoxylignan 9-O-β-D-glucopyranoside	583.2048	1.5	5.01	+HCOO	Yes	−
nonen-1-one	451.2189	0.4	5.02	+HCOO	Yes	+
Rehmannia new terpenoid A	445.2082	0.3	5.05	-H, +HCOO	No	
neo-rehmannioside	477.2351	0.9	5.1	+HCOO	Yes	−
Jioglutoside B	563.1998	1.7	5.13	+HCOO	No	
feruloyl ajugol	719.2571	1.5	5.14	-H	No	
2,4-Dimethoxy-2-methyl-6H-pyran-3-one	217.0708	−1	5.16	+HCOO	Yes	−
Rehmannia new terpenoid F	659.326	−2.4	5.19	+HCOO, -H	Yes	−
5-deoxyantirrhinoside	391.1251	0.5	5.21	+HCOO	Yes	+
Dhydrophaseic acid 4′-O-β-D-glucopyranoside	443.192	−0.2	5.29	-H	Yes	−
raffinose	623.1991	1	5.42	-H	Yes	−
Melittin monoglucoside	461.1667	0.3	5.62	-H	No	
Isoeugenol methyl ester	373.1132	−0.8	5.8	-H, +HCOO	No	
gardoside methyl ester	373.1132	−0.8	5.8	-H, +HCOO	No	
Rehmannioside A	569.1737	1.4	5.82	+HCOO	Yes	+
5-Deoxylamiol	407.1569	1.1	5.92	+HCOO	Yes	−
Jionoside B1	813.2835	1.2	5.95	-H, +HCOO	No	
Genipin 1-O-a-L-rhamnopyranosyl(1→6)-β-D-glucopyranoside	579.1984	5.3	5.96	+HCOO	No	
Carrot glycosides A	179.055	−1.1	6.01	-H	No	
1-methyl-1,2,3,4-tetrahydro-β-carboline-3-carboxylic acid	291.1358	0.8	6.04	+HCOO	No	
Cistanoside F	312.0945	−0.5	6.08	+HCOO	Yes	+
Decaffeoyl verbascoside	721.2519	−4.2	6.12	+HCOO	Yes	−
ursolic acid	393.1396	−0.6	6.22	+HCOO	NO	
Rehmannia new terpenoid J	740.2719	−2.6	6.32	+HCOO	Yes	+
manninotriose	487.1459	0.2	6.37	-H	Yes	−
Jionoside A1	799.2674	0.8	6.42	-H, +HCOO	No	
lamiol	409.1349	−0.3	6.87	+HCOO, -H	No	
Rehmanniae neoterpene C	551.2823	−3.8	6.89	-H	No	
syringic acid	407.1192	−0.3	7.13	+HCOO	No	
Genameside C	394.1133	1.6	7.13	+HCOO	Yes	+
catalpinoside	407.1192	−0.3	7.14	+HCOO	Yes	−
genipin	423.1544	3.6	7.81	+HCOO	No	
7-methylcoumarin	595.187	−0.9	7.85	+HCOO	No	
Rehmannia new terpenoid B	509.1882	0.7	7.9	-H	Yes	+
6-O-α-D-galactosyl harpagide	655.2293	4.9	8.5	-H, +HCOO	No	
glucose	569.1755	3.2	8.77	+HCOO, -H	Yes	+
Rehmannia neoterpene E	829.2788	1.6	8.84	+HCOO	Yes	−
adenosine	387.1137	−0.7	9.06	+HCOO, -H	Yes	−
Mussaenosidic acid	421.1359	0.8	9.27	+HCOO	Yes	+
Masitic acid	549.1678	0.6	9.59	+HCOO, -H	Yes	−
Rehmannioside D	731.227	1.9	9.72	+HCOO	Yes	+
melasmoside	125.0237	−0.7	9.98	-H	Yes	−
Rehmaionoside C	551.1781	1.1	10.26	+HCOO, -H	No	
stachyose	503.1614	−0.4	10.55	-H	Yes	−
verbascose	711.2204	0.3	11.1	+HCOO, -H	Yes	−
harpagoside	740.277	2.6	11.41	+HCOO	No	
Leucosceptoside A	637.2134	−0.4	11.43	-H	Yes	+
Sculponiside	735.2906	3.6	11.63	+HCOO	Yes	−
Aeginetic acid 5-O-β-D-quinovoside	595.1868	−1.2	11.85	+HCOO	No	
catapolgenin	873.2743	1.5	12.08	+HCOO, -H	No	
uridine	517.1576	1.3	13.18	+HCOO	No	

‘+’ indicates that the content of this component in RRP was greater than that in RR, and ‘−’ indicates that the content of this component in RR was greater than that in RRP.

**Table 4:** The results of docking between the active ingredient and the target molecule.

Active Ingredient Target	Binding Energy (kcal/mol)	
	AKT1	NOS3
D-Fructose	−4.5	−5.9
Glucose	−4.8	−5.5
5-hydroxymethylfurfural	−4.3	−5.3
Melibiose	−6	−7.5
Citric Acid	−4.6	−5.3
8-Epilganic Acid	−6.9	−7.5
Manninotriose	−6.1	−6.5

**Table 5:** List of primer designs.

Primers	Forward	Reverse
AKT1	AGAGGCAGGAAGAAGAGACGATGG	GCAGGACACGGTTCTCAGTAAGC
NOS3	GCAGGCATCACCAGGAAGAAGAC	CTGAGCAGGAGACACTGTTGAATCG

## Data Availability

The original contributions presented in this study are included in the article.

## Supplementary Materials

The following supporting information can be downloaded at https://www.mdpi.com/article/10.3390/ijms26083914/s1.

## Abbreviations

RP	Rehmanniae Radix
RRP	Rehmanniae Radix Praeparata
HGB	Hemoglobin
RBC	Red Blood Cells
PLT	Platelets
WBC	White Blood Cells
cAMP	Cyclic Adenosine Monophosphate
cGMP	Cyclic Guanosine Monophosphate
TCM	Traditional Chinese Medicine
BDS	Blood Deficiency Syndrome
HMDS	Hematopoietic–Metabolic Dysregulation Syndrome
LRR	Low-Dose Rehmanniae Radix Group
MRR	Middle-Dose Rehmanniae Radix Group
HRR	High-Dose Rehmanniae Radix Group
LRRP	Low-Dose Rehmanniae Radix Praeparata Group
MRRP	Middle-Dose Rehmanniae Radix Praeparata Group
HRRP	High-Dose Rehmanniae Radix Praeparata Group
APH	Acetylphenylhydrazine
CP	Cyclophosphamide
EDTA	Ethylenediaminetetraacetic Acid
ELISA	Enzyme-Linked Immunoassay
TCMSP	Traditional Chinese Medicine Systems Pharmacology Database and Analysis Platform
OMIM	Online Mendelian Inheritance in Man
KEGG	Kyoto Encyclopedia of Genes and Genomes
GO	Gene Ontology
TBST	Tween 20
BP	Biological Process
CC	Cell Composition
MF	Molecular Function
PCA	Principal Component Analysis
OPLS-DA	Orthogonal Partial Least Squares Discrimination Analysis
