# Supplementary material for: Rehmanniae Radix Praeparata in Blood Deficiency Syndrome: UPLC-Q-TOF-MS Profiling, Network Pharmacology, and PI3K-AKT Activation
Source: Int J Mol Sci. 2025 Apr 21;26(8):3914. doi: 10.3390/ijms26083914 (PMC12027966; doi:10.3390/ijms26083914)
Supplement: Supplementary file 1 [file ijms-26-03914-s001.zip › support material/Table S1.docx]

Table S1 Changes in maximum grip strength of limbs ( n = 8 )

| Group | Grip strength(N) | Recovery rate(%) |
| --- | --- | --- |
| Control | 0.62±0.07 | 100 |
| Model | 0.38±0.05** | 61.3 |
| Positive | 0.57±0.05## | 91.9 |
| LRR | 0.43±0.06# | 69.4 |
| MRR | 0.47±0.05## | 75.8 |
| HRR | 0.51±0.07## | 82.3 |
| LRRP | 0.49±0.04## | 79 |
| MRRP | 0.54±0.06## | 87.1 |
| HRRP | 0.58±0.05## | 93.5 |

Compared to the control group, the model group had P<0.05, indicated by ^#^, P<0.01; indicated by ^##^, P<0.001; indicated by ^###^, P<0.0001; and indicated by ^####^.

Compared to the model group, the remaining dosing groups had P<0.05, indicated by *, P<0.01; indicated by **, P<0.001; indicated by *** P<0.0001; and indicated by ^****^.
