# Supplementary material for: Rehmanniae Radix Praeparata in Blood Deficiency Syndrome: UPLC-Q-TOF-MS Profiling, Network Pharmacology, and PI3K-AKT Activation
Source: Int J Mol Sci. 2025 Apr 21;26(8):3914. doi: 10.3390/ijms26083914 (PMC12027966; doi:10.3390/ijms26083914)
Supplement: Supplementary file 1 [file ijms-26-03914-s001.zip › support material/Table S2.docx]

Table S2 Hair status score ( n = 8 )

| Group | 0 day | 7 day |
| --- | --- | --- |
| Control | 0.2±0.1 | 0.3±0.1 |
| Model | 2.3±0.2 | 2.4±0.3** |
| Positive | 2.2±0.1 | 0.7±0.2## |
| LRR | 2.3±0.1 | 1.9±0.3 |
| MRR | 2.2±0.2 | 1.6±0.4## |
| HRR | 2.3±0.1 | 1.3±0.3## |
| LRRP | 2.2±0.1 | 1.5±0.2## |
| MRRP | 2.3±0.2 | 0.9±0.3## |
| HRRP | 2.2±0.1 | 0.5±0.2## |

Compared to the control group, the model group had P<0.05, indicated by ^#^, P<0.01; indicated by ^##^, P<0.001; indicated by ^###^, P<0.0001; and indicated by ^####^.

Compared to the model group, the remaining dosing groups had P<0.05, indicated by *, P<0.01; indicated by **, P<0.001; indicated by *** P<0.0001; and indicated by ^****^.
