# Supplementary material for: Rehmanniae Radix Praeparata in Blood Deficiency Syndrome: UPLC-Q-TOF-MS Profiling, Network Pharmacology, and PI3K-AKT Activation
Source: Int J Mol Sci. 2025 Apr 21;26(8):3914. doi: 10.3390/ijms26083914 (PMC12027966; doi:10.3390/ijms26083914)
Supplement: Supplementary file 1 [file ijms-26-03914-s001.zip › support material/Table S3.docx]

Table S3 Dynamic changes of body weight after administration ( n = 8 )

| Group | 0 day(g) | 7 day(g) | Recovery rate(%) |
| --- | --- | --- | --- |
| Control | 34.1±0.5 | 36.3±0.6 | 100 |
| Model | 23.8±0.6 | 21.2±0.4* | 77.5 |
| Positive | 30.0±0.5 | 31.5±0.7# | 98.1 |
| LRR | 29.7±0.4 | 28.6±0.5 | 83.6 |
| MRR | 30.2±0.3 | 29.3±0.6# | 87.9 |
| HRR | 29.9±0.4 | 29.1±0.5# | 90.2 |
| LRRP | 30.1±0.6 | 28.9±0.4# | 86.4 |
| MRRP | 29.8±0.5 | 30.2±0.6# | 94.5 |
| HRRP | 30.2±0.5 | 30.8±0.7# | 97.6 |

Compared to the control group, the model group had P<0.05, indicated by ^#^, P<0.01; indicated by ^##^, P<0.001; indicated by ^###^, P<0.0001; and indicated by ^####^.

Compared to the model group, the remaining dosing groups had P<0.05, indicated by *, P<0.01; indicated by **, P<0.001; indicated by *** P<0.0001; and indicated by ^****^.
