# Supplementary material for: Rehmanniae Radix Praeparata in Blood Deficiency Syndrome: UPLC-Q-TOF-MS Profiling, Network Pharmacology, and PI3K-AKT Activation
Source: Int J Mol Sci. 2025 Apr 21;26(8):3914. doi: 10.3390/ijms26083914 (PMC12027966; doi:10.3390/ijms26083914)
Supplement: Supplementary file 1 [file ijms-26-03914-s001.zip › support material/Table S4.docx]

Table S1 Blood routine analysis between groups corresponding to the F value

|  | ​WBC | ​RGB | ​RBC | ​PLT |  |  |
| --- | --- | --- | --- | --- | --- | --- |
| ​Model vs Control | # (P=0.037, F(1,14)=7.8) | # (P=0.043, F(1,14)=7.5) | # (P=0.028, F(1,14)=8.1) | # (P=0.049, F(1,14)=7.3) |  |  |
| ​Positive vs Model | ## (P=0.005, F(1,14)=12.7) | ## (P=0.003, F(1,14)=13.1) | ## (P=0.006, F(1,14)=12.3) | ​** (P=0.009, F(1,14)=4.9) |  |  |
| ​LRR vs Model | — (P=0.11, F(1,14)=2.1) | — (P=0.15, F(1,14)=1.7) | — (P=0.08, F(1,14)=2.5) | ​** (P=0.007, F(1,14)=5.0) |  |  |
| ​MRR vs Model | — (P=0.10, F(1,14)=2.3) | — (P=0.13, F(1,14)=1.9) | * (P=0.048, F(1,14)=3.8) | — (P=0.07, F(1,14)=2.6) |  |  |
| ​HRR vs Model | — (P=0.22, F(1,14)=1.2) | — (P=0.18, F(1,14)=1.5) | — (P=0.25, F(1,14)=1.0) | — (P=0.20, F(1,14)=1.3) |  |  |
| ​LRRP vs Model | ​** (P=0.008, F(1,14)=4.7) | — (P=0.06, F(1,14)=2.7) | — (P=0.09, F(1,14)=2.2) | ​** (P=0.004, F(1,14)=5.5) |  |  |
| ​MRRP vs Model | — (P=0.14, F(1,14)=1.8) | * (P=0.032, F(1,14)=3.7) | * (P=0.041, F(1,14)=3.5) | ​** (P=0.001, F(1,14)=6.1) |  |  |
| ​HRRP vs Model | ​** (P=0.002, F(1,14)=5.9) | ​** (P=0.001, F(1,14)=6.3) | ​** (P=0.003, F(1,14)=5.7) | ​** (P<0.001, F(1,14)=7.8) |  |  |
