# Supplementary material for: Rehmanniae Radix Praeparata in Blood Deficiency Syndrome: UPLC-Q-TOF-MS Profiling, Network Pharmacology, and PI3K-AKT Activation
Source: Int J Mol Sci. 2025 Apr 21;26(8):3914. doi: 10.3390/ijms26083914 (PMC12027966; doi:10.3390/ijms26083914)
Supplement: Supplementary file 1 [file ijms-26-03914-s001.zip › support material/Table S5.docx]

Table S5 cAMP and cGMP analysis between groups corresponding to the F value

|  | ​cAMP | ​cGMP |
| --- | --- | --- |
| ​Model vs Control | ## (P=0.003, F(1,14)=13.2) | ## (P=0.002, F(1,14)=13.8) |
| ​Positive vs Model | ​** (P=0.008, F(1,14)=4.9) | ​** (P=0.007, F(1,14)=5.1) |
| ​LRR vs Model | — (P=0.15, F(1,14)=1.8) | * (P=0.04, F(1,14)=3.5) |
| ​MRR vs Model | ​** (P=0.009, F(1,14)=4.7) | * (P=0.03, F(1,14)=3.2) |
| ​HRR vs Model | ​** (P=0.006, F(1,14)=5.3) | * (P=0.02, F(1,14)=3.8) |
| ​LRRP vs Model | — (P=0.12, F(1,14)=2.1) | — (P=0.07, F(1,14)=2.4) |
| ​MRRP vs Model | ​** (P=0.005, F(1,14)=5.5) | ​** (P=0.004, F(1,14)=5.7) |
| ​HRRP vs Model | ​** (P=0.001, F(1,14)=6.2) | ​** (P<0.001, F(1,14)=7.1) |
