# Supplementary figures and images for: Rehmanniae Radix Praeparata in Blood Deficiency Syndrome: UPLC-Q-TOF-MS Profiling, Network Pharmacology, and PI3K-AKT Activation
Source: Int J Mol Sci. 2025 Apr 21;26(8):3914. doi: 10.3390/ijms26083914 (PMC12027966; doi:10.3390/ijms26083914)

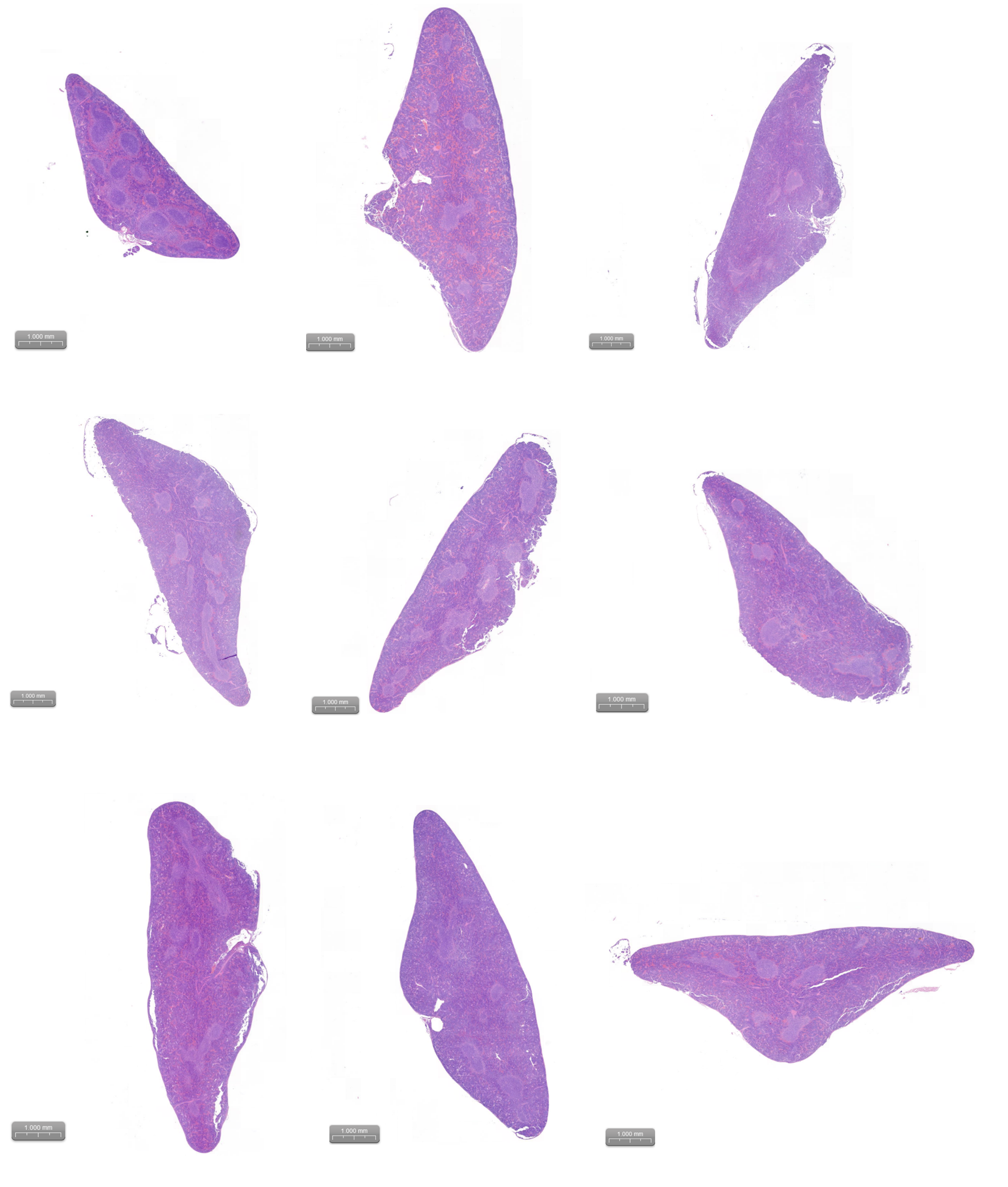

Supplement: Supplementary file 1 [file ijms-26-03914-s001.zip › support material/Fig. S1.tif]

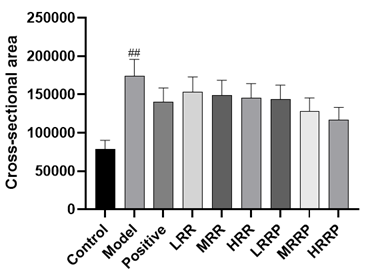

Supplement: Supplementary file 1 [file ijms-26-03914-s001.zip › support material/Fig. S2.tif]

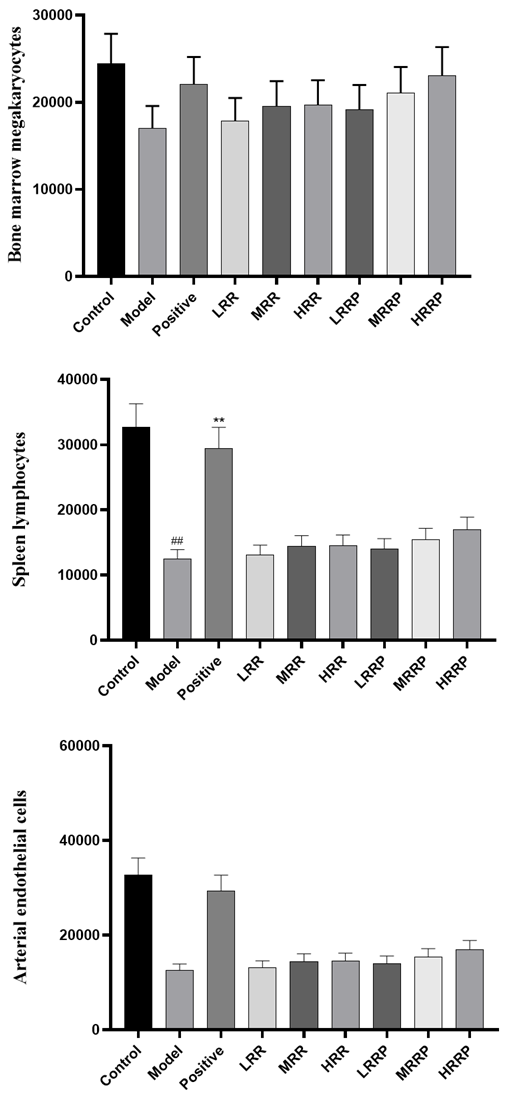

Supplement: Supplementary file 1 [file ijms-26-03914-s001.zip › support material/Fig. S3.tif]

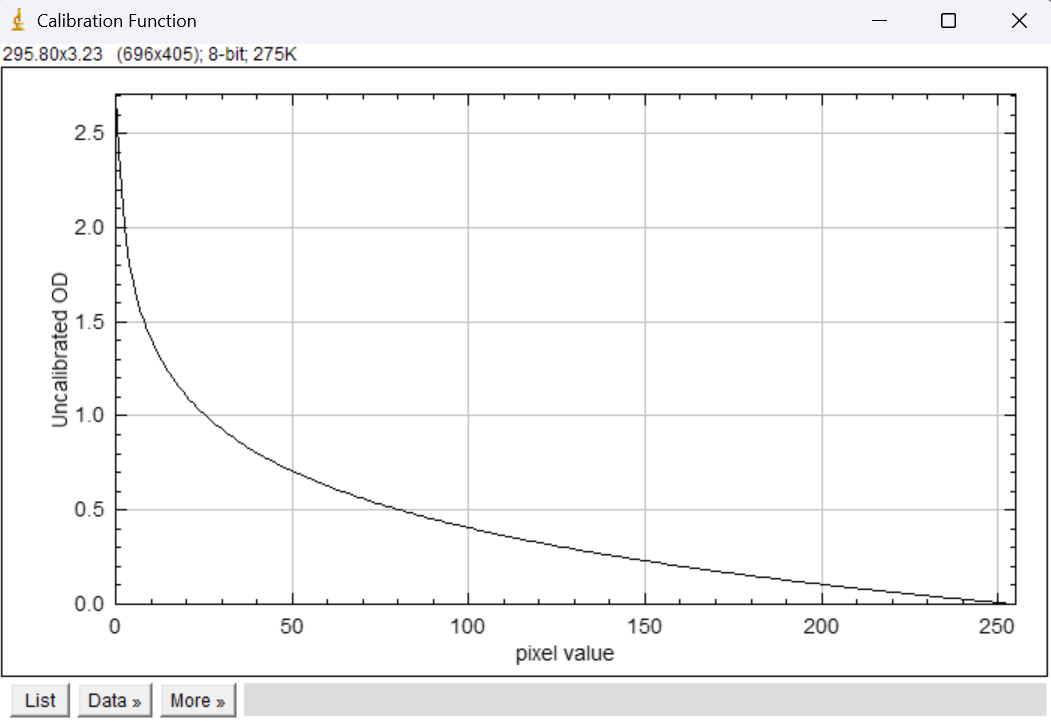

Supplement: Supplementary file 1 [file ijms-26-03914-s001.zip › support material/Fig. S4.png]
